# Response strategies of fine root morphology of *Cupressus funebris* to the different soil environment

**DOI:** 10.3389/fpls.2022.1077090

**Published:** 2022-12-21

**Authors:** Xiaochen Wen, Xiao Wang, Mengting Ye, Hai Liu, Wenchun He, Yu Wang, Tianyi Li, Kuangji Zhao, Guirong Hou, Gang Chen, Xianwei Li, Chuan Fan

**Affiliations:** ^1^ College of Forestry, Sichuan Agricultural University, Chengdu, China; ^2^ National Forestry and Grassland Administration Key Laboratory of Forest Resources Conservation and Ecological Safety on the Upper Reaches of the Yangtze River and Forestry Ecological Engineering in the Upper Reaches of the Yangtze River Key Laboratory of Sichuan Province, Chengdu, China

**Keywords:** fine root, morphological plasticity, root order, response strategy, soil environment, *Cupressus funebris*

## Abstract

Understanding fine root morphology is crucial to uncover water and nutrient acquisition and transposition of fine roots. However, there is still a lack of knowledge regarding how the soil environment affects the fine root morphology of various root orders in the stable forest ecosystem. Therefore, this experiment assessed the response strategies of fine root morphology (first- to fifth -order fine roots) in four different soil environments. The results showed that fine root morphology was related to soil environment, and there were significant differences in specific root length (SRL), specific surface area (SRA), diameter (D), and root tissue density (RTD) of first- and second -order fine roots. Soil total nitrogen (TN), alkaline nitrogen (AN) and available phosphorus (AP) were positively correlated with SRL and SRA and negatively correlated with D and RTD. Soil moisture (SW) was positively correlated with the D and RTD of first- and second-order fine roots and negatively correlated with the SRL and SRA. Soil temperature (ST), organic carbon (OC), soil bulk density (SBD) and soil porosity (SP) were not significantly correlated with the D, SRL, SRA, and RTD of the first- and second -order fine roots. AN was positively correlated with SRL and SRA and negatively correlated with both D and RTD in the first- and second -order fine roots, and the correlation coefficient was very significant. Therefore, we finally concluded that soil AN was the most critical factor affecting root D, SRL, SRA and RTD of fine roots, and mainly affected the morphology of first- and second -order fine roots. In conclusion, our research provides support for understanding the relationship between fine root morphology and soil environment, and indicates that soil nutrient gradient forms good root morphology at intraspecific scale.

## Introduction

The fine roots were the main tissue for nutrient and water acquisition underground, as well as the most active part of nutrient absorption and transport (Bardgett et al., 2014; McCormack et al., 2015). Fine roots regulated nutrient cycling in forest ecosystems and were highly plastic to soil nutrient availability (Hodge et al., 2009). Nevertheless, fine root morphology affected its function. For instance, a smaller root diameter (D) had higher metabolic activity, and its moisture absorption capacity was stronger (Montagnoli, 2018). The D, specific root length (SRL), specific surface area (SRA), and root tissue density (RTD) of fine roots were important morphological indicators determining plant growth (Reich, 2014; Roumet et al., 2016; Freschet et al., 2021). These morphological indicators played an important role in obtaining water and nutrient resources (Bardgett et al., 2014; Iversen et al., 2017). For example, the increase of SRL and SRA in fine roots promoted the ability of plants to obtain resources (Weemstra et al., 2016a; Addo-Danso et al., 2018). The change in fine root morphology is influenced by external factors such as soil fertility (Eissenstat et al., 2000). Thus, the study of fine root morphology can help us understand how fine roots adapt to their surroundings.

There were currently several discussions on fine root morphology adaptation strategies in different soil environments. The fine root morphology changed and took on an asymmetrical distribution in the different soil environments (Yan et al., 2019). For instance, fine roots grew longer in dry areas than they did in moist areas (Bakker et al., 2006). The theory of resource economics stated that SRA and SRL had a direct connection to nutrient and moisture absorption (Hertel et al., 2013). Fine root SRL and SRA growth could improve access to resources (Verburg et al., 2013; Weemstra et al., 2016b). Fine root turnover was increased in warm soils, and fine root SRL and SRA considerably rose (Kengdo et al., 2022). Plants would alter the formation of fine roots in areas with high soil fertility in order to swiftly access nutrients (Fransen and de Kroon, 2001). While it had also been constricted that heavy fertilizer administration slowed fine root growth, plants in nutrient-rich environments lengthened their fine roots and shortened their fine root SRL in order to acquire more nutrients (Yang et al., 2021; Zhu et al., 2021). According to Pérez-Ramos et al. (2012), fine roots in poor soils could increase the length, RTD, and number of fine roots, or decrease SRA and increase RTD to maintain growth (Lõhmus et al., 2006). Meanwhile, the architecture of fine roots allowed trees to adjust to normal growth in harsh situations (Ostonen et al., 2007). For instance, in drought-prone environments, plants could increase the quantity of fine roots or modify D and RTD variations (Tan et al., 2021; De Andrade et al., 2022). In response to moisture stress, fine roots would increase RTD and decrease D and SRA (Hertel et al., 2013; Wurzburger and Wright, 2015; Nikolova et al., 2020). In order to adjust to the soil environment, trees would grow fine roots D and decrease SRL and SRA in cold environments (Zadworny et al., 2017; Defrenne et al., 2019). In high soil bulk density (SBD), fine roots would increase D to resist the solid soil environment (Clark et al., 2003).

The morphology and function of fine roots in different root orders were significantly different, and they also responded differently to the soil environment (Guo et al., 2008; Reich, 2014). However, the traditional definition of sampling (diameter less than 2 mm) was used to judge the effect of fine roots on nutrient cycling (Wells and Eissenstat, 2001; Finér et al., 2011). Although this standard had differences in structure, physiology, and morphology, it ignored the differences in fine root structure and internal function between different root orders (Pregitzer et al., 2002). For example, nitrogen and phosphorus content and fine root structure might vary in the root D of different tree species (Li et al., 2010; Wang et al., 2022). The division of fine roots based root order could weaken the internal heterogeneity of fine roots and more effectively descdribe the physiological processes of fine roots of different root orders (Guo et al., 2008). Therefore, dividing fine root morphology based different root orders could better reflect root function and nutrient dynamics (Guo et al., 2008; Fan and Jiang, 2010; Mei et al., 2010). To grade roots, the shaft distal end of a root with no branching root was the first order, and first-order fine roots were derived from second-order fine roots, while second-order fine roots were derived from third-order fine roots, and so on up to fifth-order fine roots (Pregitzer et al., 2002). However, the current research has focused on the absorbent roots (first and second or third fine roots), and the morphological research of first- to fifth-order fine roots was still rare. For example, some studies have looked into low order roots (first- and second -order fine roots) in temperate forests. The relationship between apical (first –order fine roots) morphology and environment has also been discussed (Liese et al., 2017; Defrenne et al., 2019; Ugawa et al., 2022). Therefore, studying the relationship between fine root morphology and different soil environments from the perspective of root order was of great significance for understanding the heterogeneity within the root system.

However, previous studies on plant fine root morphology were mostly manipulation experiments or only investigated the influence of a single factor (McCormack et al., 2020; Suseela et al., 2020), and few studied on multi factors under stable forest ecosystem (Leuschner et al., 2004; Valverde-Barrantes et al., 2007; Hertel et al., 2013). For example, fine roots have morphological response to nitrogen fertilizer addition (Zhu et al., 2021; Xiao et al., 2022). The study of forest ecosystems in a stable state was closer to the actual situation of plant physiological growth. Therefore, it was necessary to explore the multivariate study of fine root morphology in stable forest ecosystems.

*Cupressus funebris* was a common and widely distributed evergreen conifer tree in the middle and upper reaches of the Yangtze River in China (Wang et al., 2021). Therefore, four study sites with *C. funebris* plantations under natural conditions were selected in Guang’an (GA), a low mountainous area; Deyang (DY), a middle hill area; Suining (SN), a shallow hill area; and Mianyang (MY), a high hill area, in northeast Sichuan, China. We mainly studied the relationship between the first- to fifth -order fine root morphology (D, SRL, SRA, and RTD) and different soil environments. Two hypotheses were proposed in this study: (1) fine root orders have different morphological plasticity to soil environment and (2) there should be dominant factors influencing fine root morphology in soil environment.

## Materials and methods

### Study site

**Figure 1:**
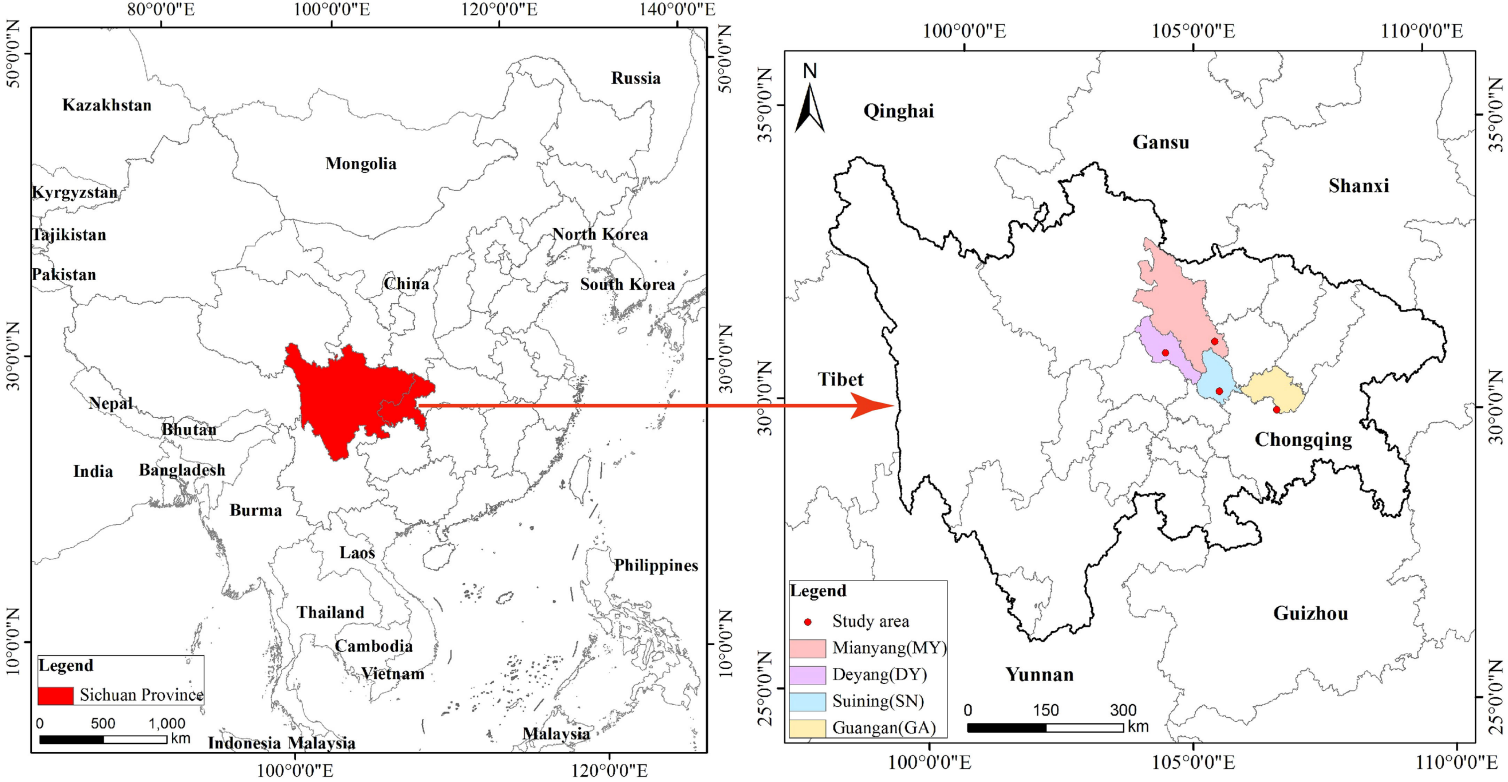
Location of the four research sites. Four study area in Sichuan province Mianyang (MY), Deyang (DY), Suining (SN), Guangan (GA).

According to the main areas of distribution of *C. funebris* plantations in the Sichuan Basin and based on a literature review and field investigation, four representative sites in northeast Sichuan Province, China, were established. The four research sites were GA, DY, MY and SN City, which respectively represented low mountain, middle hill, shallow hill and high hill areas (**Figure 1**). All sites were in a subtropical monsoon climate zone, and all *C. funebris* trees were aged 25 to 30 years. Undergrowth vegetation included primarily *Myrsine africana*, *Vitex negundo*, *Coriaria nepalensis*, *Smilax china*, *Oplismenus compositus*, *Carex brunnea*, *Cyperus rotundus*, *Ficus tikoua*, *Adiantum capillus-veneris*, *Parathelypteris glanduligera*, and *Pteris cretica*. Additional information on the research sites was provided in **Table 1**.

**Table 1:** Geographic information on different research sites.

Item	Sites			
	Guangan	Deyang	Mianyang	Suining
Latitude	106°41′27″	105°26′39″	105°31′57″	104°25′41″
Longitude	30°04′58″	31°15′54″	30°24′37″	31°04′01″
Mean annual precipitation (mm)	1150	1150	880	930
Mean annual temperature (°C)	15.7°	16.5°	17.3°	17.4°
Altitude (m)	939	415	378	530
Slope gradient (°)	28°	27°	25°	23°
Slope aspect	Southeast	Southwest	Southeast	Southwest
Soil type	Weakly acidic soil	Weakly alkaline soil	Alkaline soil	Alkaline soil
Stand age (y)	25–30	25–30	25–30	25–30
Stand density (a. h^−1^)	1,515	1,470	1,695	1,740
Crown density	0.7	0.7	0.8	0.8
Average tree height (m)	6.5	9.3	7.5	8.4
Average DBH (cm)	8.8	12.6	11.5	12.1

a, one plant. DBH, average diameter at breast height.

### Field sampling

In May 2019, forest subconditions (density and age, among other characteristics) were recorded at each site, which were consistent with little disturbance and well-developed natural vegetation, and standard lands were identified for forest subsites at each research site. The soil depth at the sample sites was approximately 30 cm. The standard plot size was 20 m × 30 m, and three plots were set up at each of the four sites for a total of 12 plots. In July 2019, three average *C. funebris* trees (trees of approximately the same average diameter at breast height (DBH), height, and mean diameter) were selected in each plot, and surface vegetation was removed 1 to 1.5 m from each trunk base. In four directions in the southeast and northwest, 20 cm × 20 cm × 20 cm blocks of soil were excavated, and surface soil and other impurities were carefully removed. We first determine the position of the target tree taproots according to its growth direction, and collect root samples. Then we combine the color (purple red), skin texture, smell (*C. funebris* essential oil), elasticity and the ease of root separation from the central column to put the live *C. funebris* roots into the fine sieve and remove other plant roots. Finally, we put the complete root segment of *C. funebris* with five orders of fine roots in the collection bags. Simultaneously with root collection, soil samples for determination of chemical composition were collected near each standard root sampling point to a depth consistent with that of root collection (0–20 cm). The diameter of the soil drill was 5 cm. To measure SBD and soil porosity (SP), soil samples at 0–20 cm were also collected with a 100 cm^3^ ring knife. We put the roots and soil in a self-sealing bag, stored them in the refrigerator, and then took them back to the lab.

### Measurement of fine root morphology

Soil attached to fine roots was removed with low-temperature deionized moisture in the laboratory, and then roots were put in a 15-cm-diameter Petri dish containing low-temperature deionized moisture (2-4°C). Fine roots were graded according to the method of Pregitzer et al. (2002). First-order fine roots were derived from second-order fine roots, second-order fine roots were derived from third-order fine roots, and so on up to fifth-order fine roots. In addition, single roots attached to higher-order fine roots were also classified as first-order fine roots (**Figure S1**; Pregitzer et al., 2002). Tweezers were used to remove each order of roots, which were put into a Petri dish containing a mixture of ice and moisture, and the root number was recorded. An Epson digital scanner (Expression 10000XL 1.0; Epson, Suwa City, Japan) was used to scan roots, and root image analysis system software (Win RHIZO Pro2009c; Canadian Ward Precision (Beijing) Technology Trading Co., Ltd., Beijing, China). It was used to analyze the morphological characteristics of fine roots of different root orders, including root D, length, surface area, and volume. After roots were scanned, they were oven-dried at 65°C to a constant weight, and dry mass was determined. Data on fine root D, length, surface area, and volume measured according to root image analysis system software were combined with fine root dry weights to calculate SRL, SRA, RTD and D (direct measurement). The calculation formula is as follows:


(1) $$SRL\;\left(m\;g^{-1}\right)=\frac{L}{M}$$

(2) $$SRA\;\left(cm^{2}\;g^{-1}\right)=\frac{S}{M}$$

(3) $$RTD\;\left(g\;cm^{-3}\right)=\frac{M}{V}$$

(4) $$CR\;\left(\%\right)=\frac{\left(Max-Min\right)}{Min}\times 100\%$$

Where *L* is the root length, *M* is the root dry weight, *S* is the root surface area, *V* is the root volume, *CR* is the change rate, *Max* is the maximum value, and *Min* is the minimum value.

### Soil physicochemical properties

At each standard point, soil moisture (SW) was measured by drying (Schmugge et al., 1980). Organic carbon (OC) was measured by a potassium dichromate oxidation–external heating method (Sato et al., 2014). Alkaline nitrogen (AN) was determined by an alkali-diffusion method (Keeney and Bremner, 1966). Available phosphorus (AP) was determined by sodium bicarbonate extraction (Li et al., 2010). Total nitrogen (TN) was determined by a semi-micro Kjeldahl method (Trikilidou et al., 2020) and total phosphorus (TP) was determined by an alkali fusion-molybdenum antimony colorimetric method (Chapin, 1983). ST was measured with a button thermometer (DS1921G; Shanghai Bobamban Electronic Technology Co., Ltd., Shanghai, China). In May 2019, a thermometer was buried at a 10-cm depth in the soil at each site and set to record every 2 hours, and data were read when root systems were sampled (Lee and Wang, 2017). SBD and SP were measured using the tangent loop method (Chen et al., 2021).

### Statistical analyses

Data were analyzed by SPSS 20.0 software (SPSS Inc., Chicago, IL, United States). Effects of root order and location on the morphological characteristics of fine roots were analyzed by two-way ANOVA, whereas the morphological characteristics of fine roots and differences in soil physicochemical properties were analyzed by one-way ANOVA. The least significant difference (LSD) method was used for multiple comparisons, with significant differences at *P<* 0.05. Pearson correlations were used to examine relations between different soil indices and root order morphology. Redundancy analysis (RDA) of fine root morphological characteristics and soil environmental factors was conducted with Canoco software (version 5.0) and get contribution rate. For all statistical tests, the significance level was 0.05. The figure was produced using RStudio (version 4.1.0).

## Results

### Effect of soil physical and chemical properties on fine root morphology of *Cupressus funebris*

The SRL, SRA, D and RTD of fine roots were significantly affected by root order and site (*P<* 0.05). However, the interaction effect between root order and site on fine root indices was not significant (*P* > 0.05; **Table S1**). In the four research sites, differences in SRL, SRA, D and RTD of fine roots were observed primarily in first- and second -order fine roots, and differences in third- to fifth -order fine roots were not significant (**Table 2**). The values of the fine root morphology differed at the four research sites. We calculated its change rate using Formula 4. Under the influence of different soil environments, the SRL change rate of first -order fine roots was 53%, the SRA change rate was 20%, the D change rate was 23%, and the RTD change rate was 29%, while the SRL change rate of second -order fine roots was 36%, the SRA change rate was 21%, the D change rate was 26%, and the RTD change rate was 27% (**Table 2**). Among the four sites, there were no significant differences in soil TP content, but there were significant differences in AN, AP, OC, and TN contents, and SW, ST, SBD, and SP (**Table S2**). After difference significance analysis (LSD), the four sites showed significant differences in first- and second -order fine root morphology, while there was no difference in third- to fifth -order fine roots (**Table 2**). This indicated that third- to fifth -order fine root morphology was not affected by the physical and chemical properties of the soil. Therefore, we further analyzed the correlation of soil AN, TN, AP, OC, SW, SBD, ST, SP, and first- and second -order fine root morphology.

**Table 2:** Differences in characteristics of *Cupressus funebris* fine root morphology in different root orders at four research sites.

		Site			
Morphology of fine roots	Order	GA	DY	SN	MY
	1	0.54 ± 0.02c	0.44 ± 0.02a	0.52 ± 0.01c	0.47 ± 0.02b
	2	0.67 ± 0.02b	0.53 ± 0.01a	0.65 ± 0.02b	0.56 ± 0.01a
D	3	0.93 ± 0.09a	0.83 ± 0.12a	0.90 ± 0.08a	0.84 ± 0.02a
(mm)	4	1.27 ± 0.09a	1.17 ± 0.13a	1.25 ± 0.08a	1.22 ± 0.06a
	5	1.70 ± 0.11a	1.61 ± 0.10a	1.69 ± 0.13a	1.67 ± 0.19a
	1	16.21 ± 0.26b	20.78 ± 2.07c	13.54 ± 1.33a	17.71 ± 2.95b
	2	12.42 ± 1.13b	14.02 ± 1.07b	10.28 ± 0.47a	13.24 ± 0.49b
SRL	3	5.53 ± 0.80a	6.26 ± 0.86a	5.50 ± 0.54a	5.79 ± 0.50a
(m g^−1^)	4	2.88 ± 0.35a	2.92 ± 0.46a	2.58 ± 0.15a	2.92 ± 0.37a
	5	1.05 ± 0.20a	1.14 ± 0.27a	1.24 ± 0.11a	1.14 ± 0.18a
	1	264.84 ± 9.41a	301.03 ± 14.74b	250.48 ± 8.87a	286.28 ± 9.16b
	2	235.57 ± 4.43b	266.28 ± 11.61c	220.08 ± 8.54a	252.14 ± 6.31c
SRA	3	149.85 ± 10.40a	162.08 ± 17.34a	148.82 ± 18.04a	154.18 ± 7.19a
(cm^2^ g^−1^)	4	97.18 ± 10.59a	98.21 ± 19.98a	96.45 ± 3.08a	97.22 ± 7.24a
	5	57.97 ± 3.85a	62.23 ± 13.50a	56.90 ± 4.41a	59.73 ± 7.17a
	1	0.28 ± 0.01b	0.24 ± 0.01a	0.31 ± 0.02c	0.26 ± 0.01a
	2	0.31 ± 0.01b	0.26 ± 0.00a	0.33 ± 0.14b	0.28 ± 0.01a
RTD	3	0.34 ± 0.01a	0.32 ± 0.02a	0.34 ± 0.02a	0.33 ± 0.03a
(g cm^−3^)	4	0.37 ± 0.38a	0.35 ± 0.02a	0.38 ± 0.05a	0.36 ± 0.02a
	5	0.43 ± 0.04a	0.43 ± 0.21a	0.45 ± 0.02a	0.43 ± 0.03a

D, diameter; SRL, specific root length; SRA, specific surface area; RTD, root tissue density; GA, Guangan; DY, Deyang; SN, Suining; MY, Mianyang. Different lowercase letters represent significant differences in the same order at different sites (P < 0.05).

**Figure 2:**
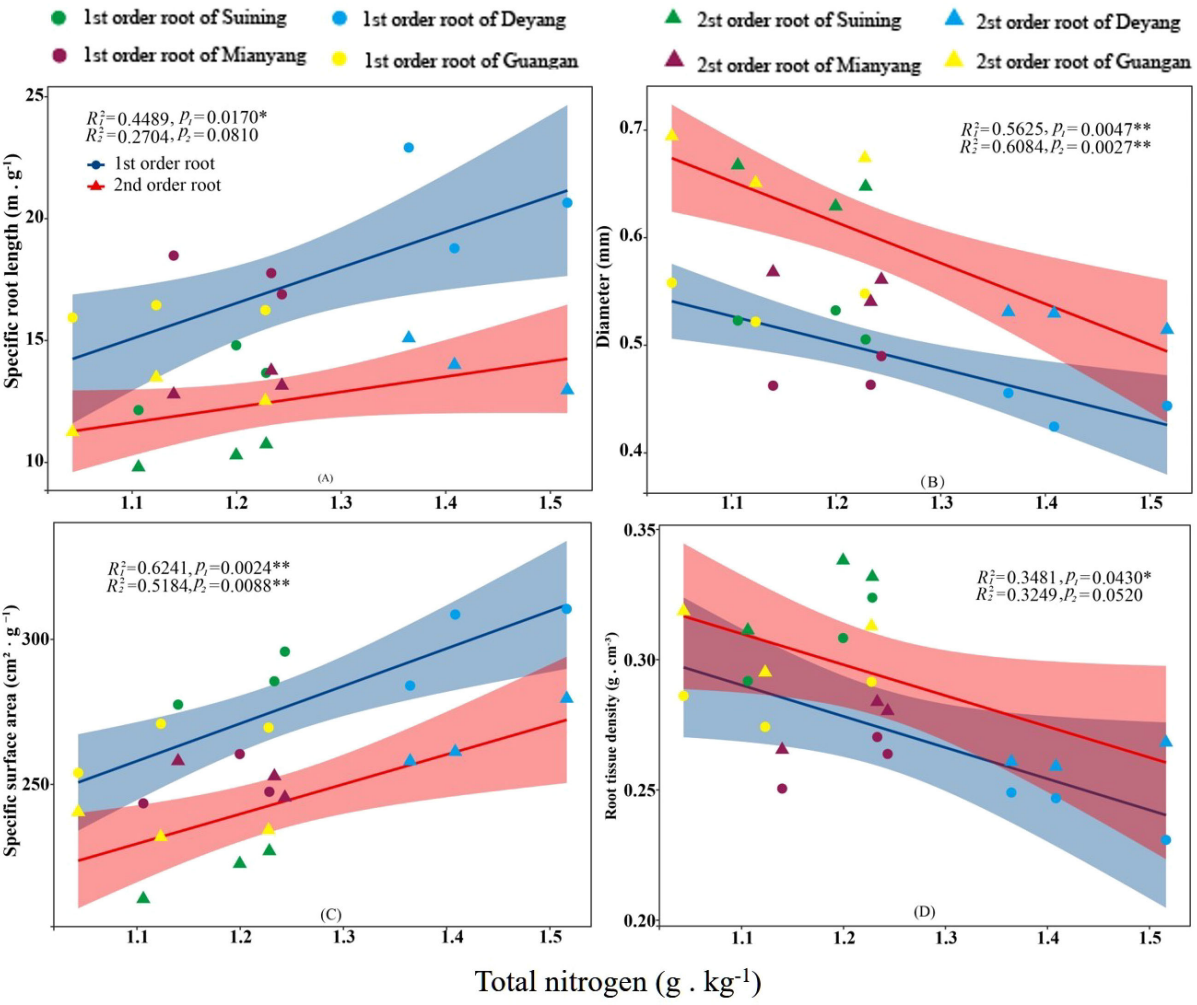
Effects of soil total nitrogen on specific root length, root diameter, specific root surface area, and root tissue density at four research sites. *R*_1_^2^ and *R*_2_^2^ represent coefficients of determination for regressions of first- and second-order fine roots *and **indicate significance at *p* < 0.05 and *p* < 0.01, respectively. *p*_1_ and *p*_2_ are *p-*values indicating significance of regressions with first- and second-order fine roots, respectively. The shaded part is the confidence interval of the fitting line, regimes (n = 3 per treatment).

**Figure 3:**
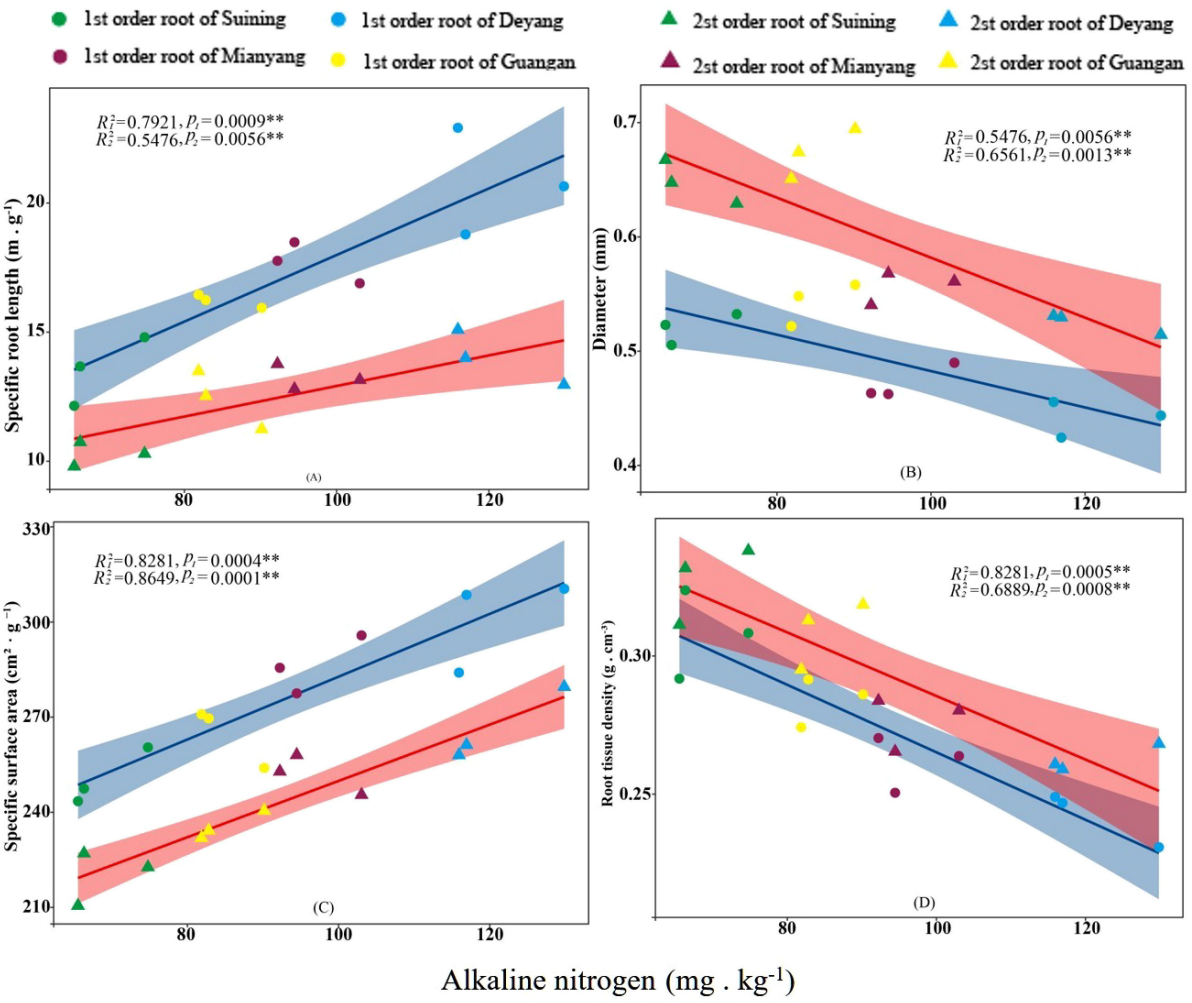
Effects of soil alkaline nitrogen on specific root length, root diameter, specific root surface area, and root tissue density at four research sites. *R*_1_^2^ and *R*_2_^2^ represent coefficients of determination for regressions of first- and second-order fine roots *and **indicate significance at *p* < 0.05 and *p* < 0.01, respectively. *p*_1_ and *p*_2_ are *p-*values indicating significance of regressions with first- and second-order fine roots, respectively. The shaded part is the confidence interval of the fitting line, regimes (n = 3 per treatment).

**Figure 4:**
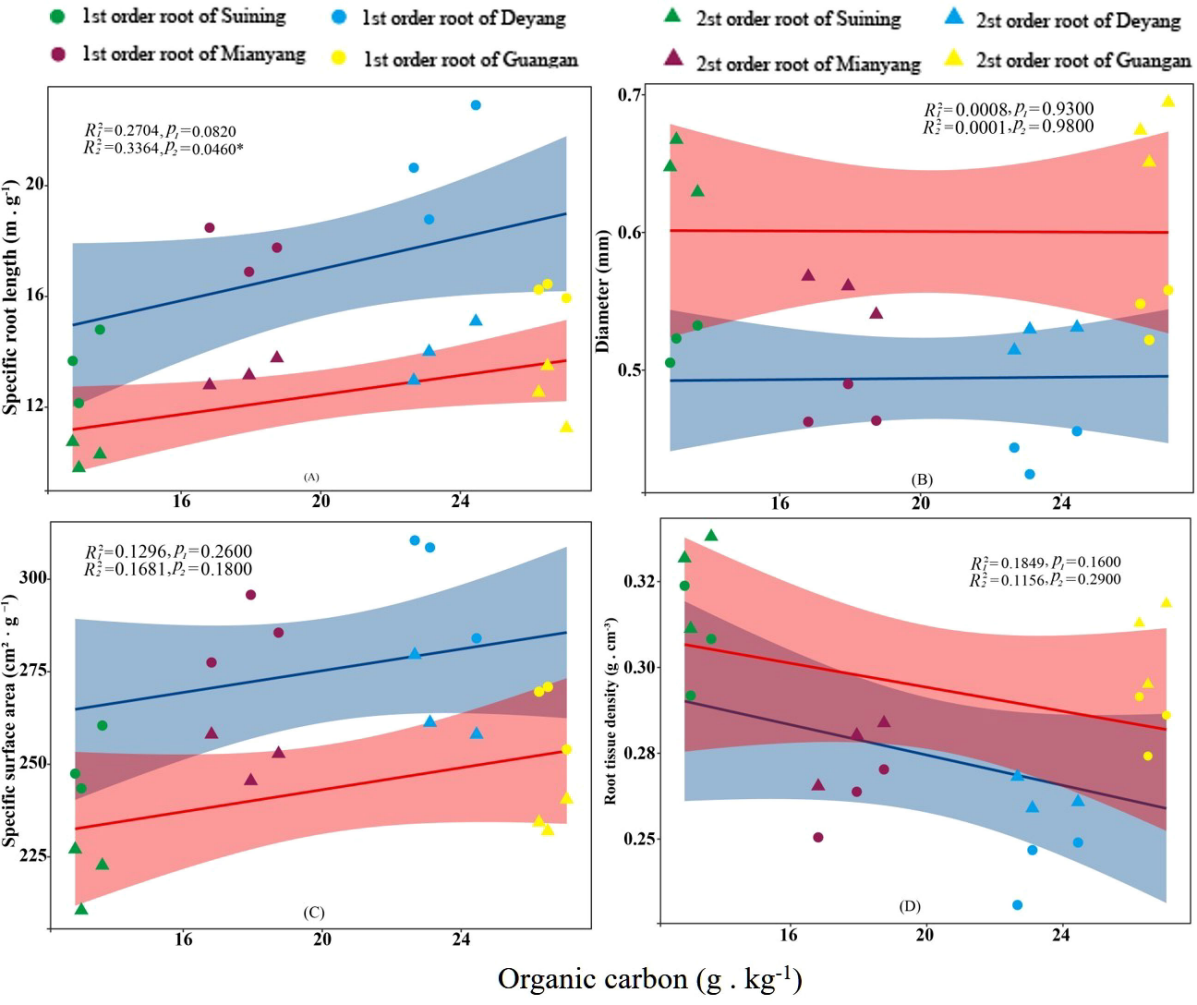
Effects of soil organic carbon on specific root length, root diameter, specific root surface area, and root tissue density at four research sites. *R*_1_^2^ and *R*_2_^2^ represent coefficients of determination for regressions of first- and second-order fine roots *and **indicate significance at *p* < 0.05 and *p* < 0.01, respectively. *p*_1_ and *p*_2_ are *p-*values indicating significance of regressions with first- and second-order fine roots, respectively. The shaded part is the confidence interval of the fitting line, regimes (n = 3 per treatment).

**Figure 5:**
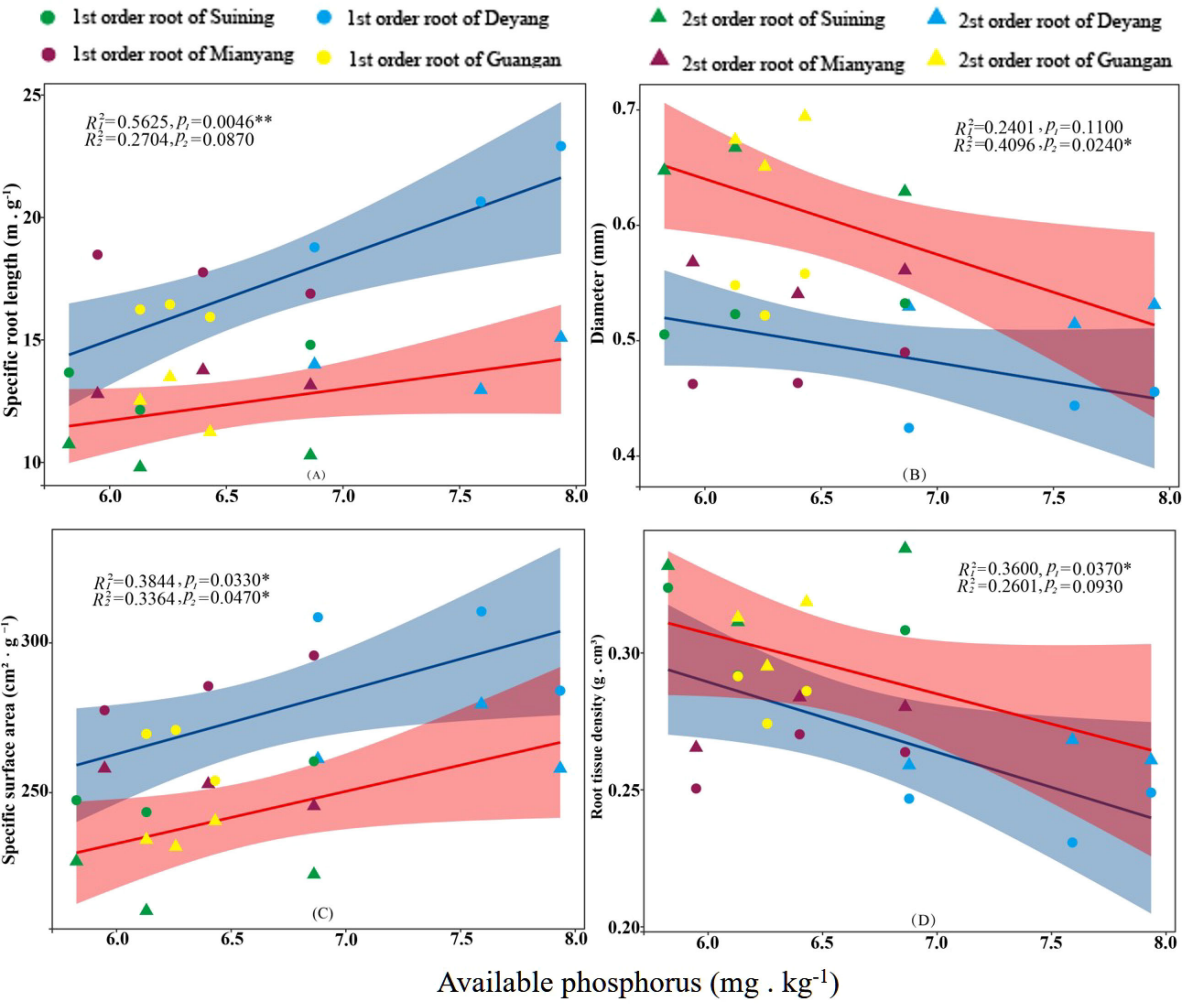
Effects of soil available phosphorus on specific root length, root diameter, specific root surface area, and root tissue density at four research sites. *R*_1_^2^ and *R*_2_^2^ represent coefficients of determination for regressions of first- and second-order fine roots *and **indicate significance at *p* < 0.05 and *p* < 0.01, respectively. *p*_1_ and *p*_2_ are *p-*values indicating significance of regressions with first- and second-order fine roots, respectively. The shaded part is the confidence interval of the fitting line, regimes (n = 3 per treatment).

**Figure 6:**
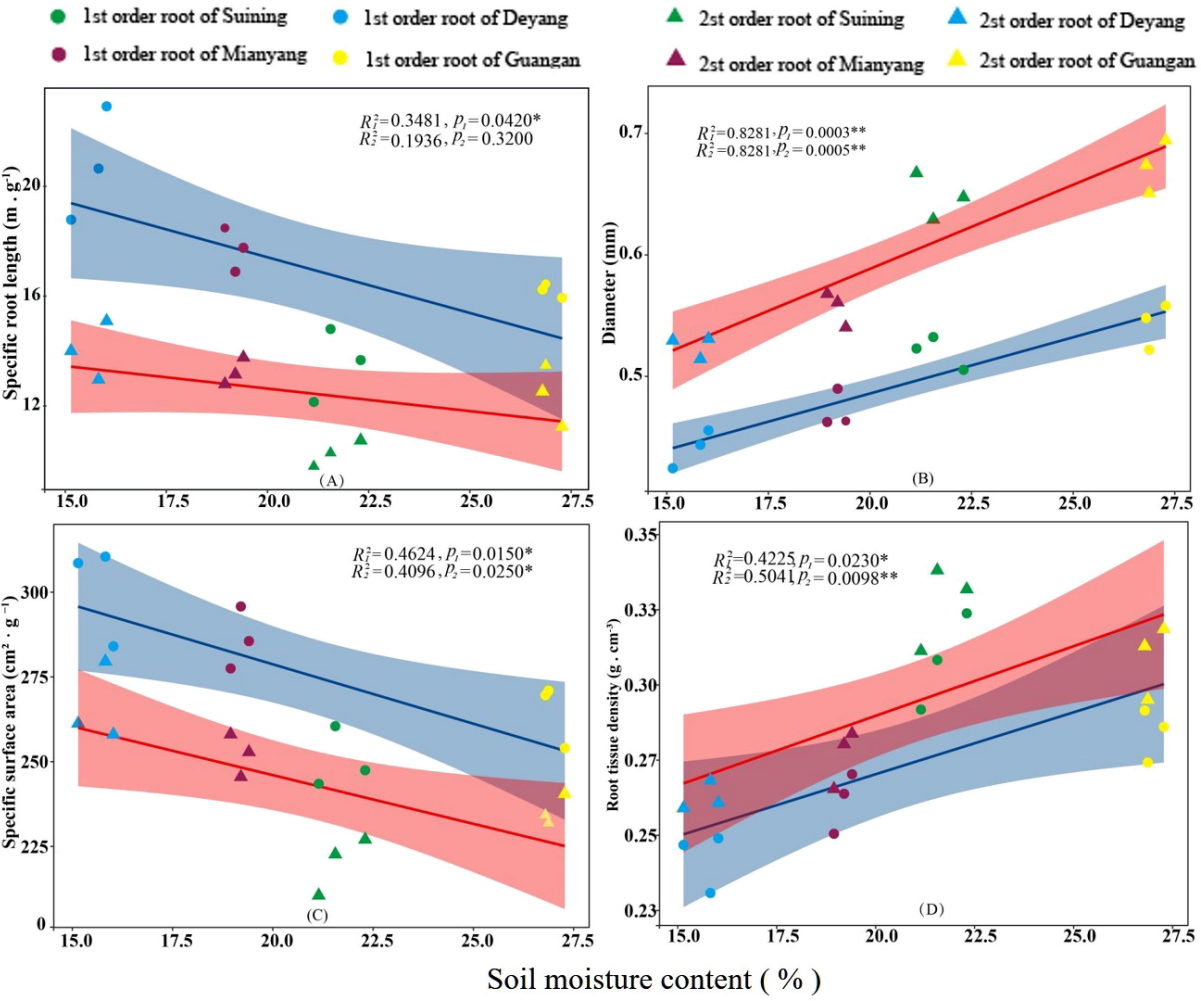
Effects of soil moisture on specific root length, root diameter, specific root surface area, and root tissue density at four research sites. *R_1_^2^* and *R_2_^2^* represent coefficients of determination for regressions of first- and second-order fine roots * and ** indicate significance at *p* < 0.05 and *p* < 0.01, respectively. *p*_1_ and *p*_2_ are p-values indicating significance of regressions with first- and second-order fine roots, respectively. The shaded part is the confidence interval of the fitting line, regimes (n = 3 per treatment).

**Figure 7:**
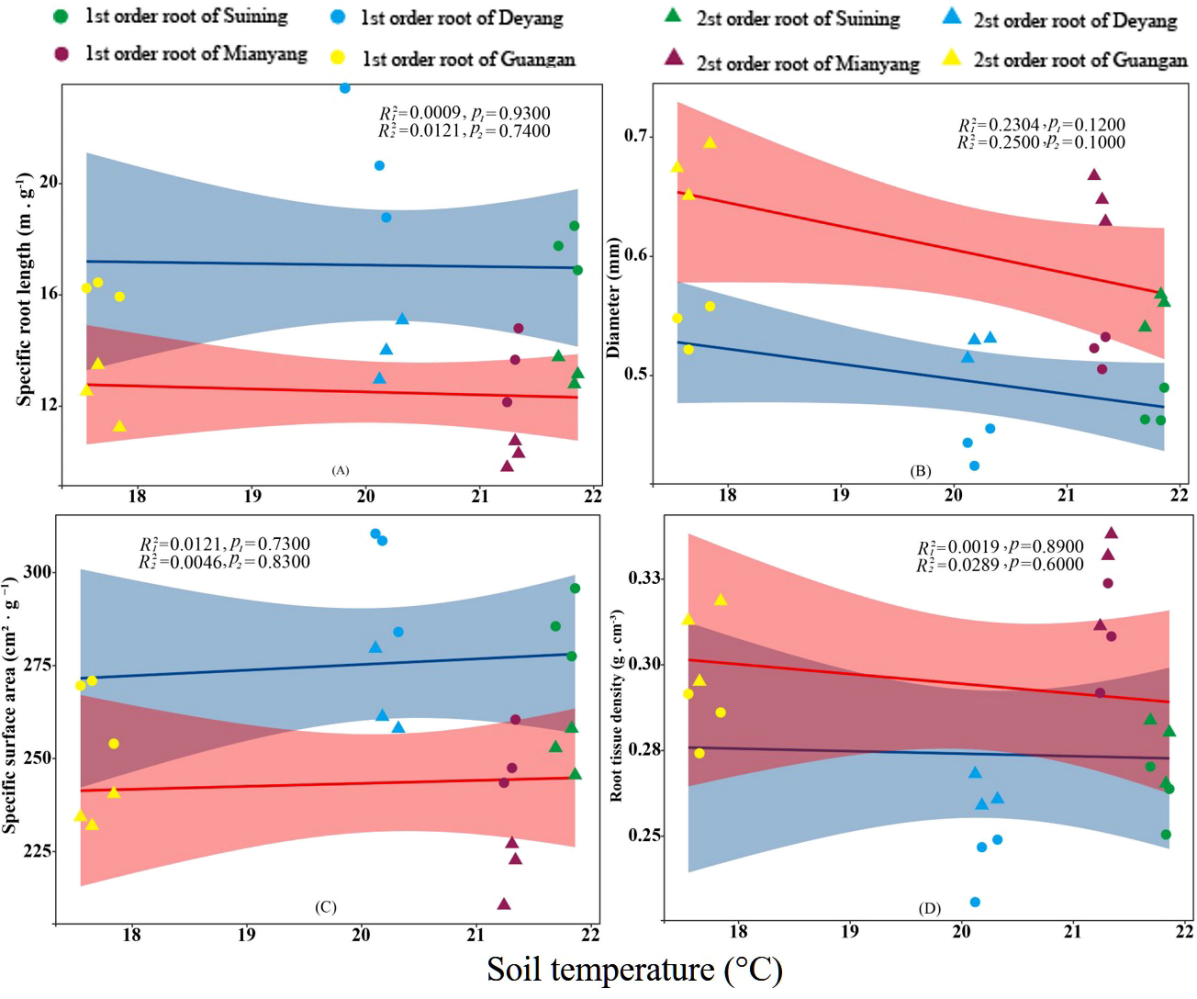
Effects of soil temperature specific root length, root diameter, specific root surface area, and root tissue density at four research sites. *R_1_^2^* and *R_2_^2^* represent coefficients of determination for regressions of first- and second-order fine roots * and ** indicate significance at *p* < 0.05 and *p* < 0.01, respectively. *p*_1_ and *p*_2_ are p-values indicating significance of regressions with first- and second-order fine roots, respectively. The shaded part is the confidence interval of the fitting line, regimes (n = 3 per treatment).

**Figure 8:**
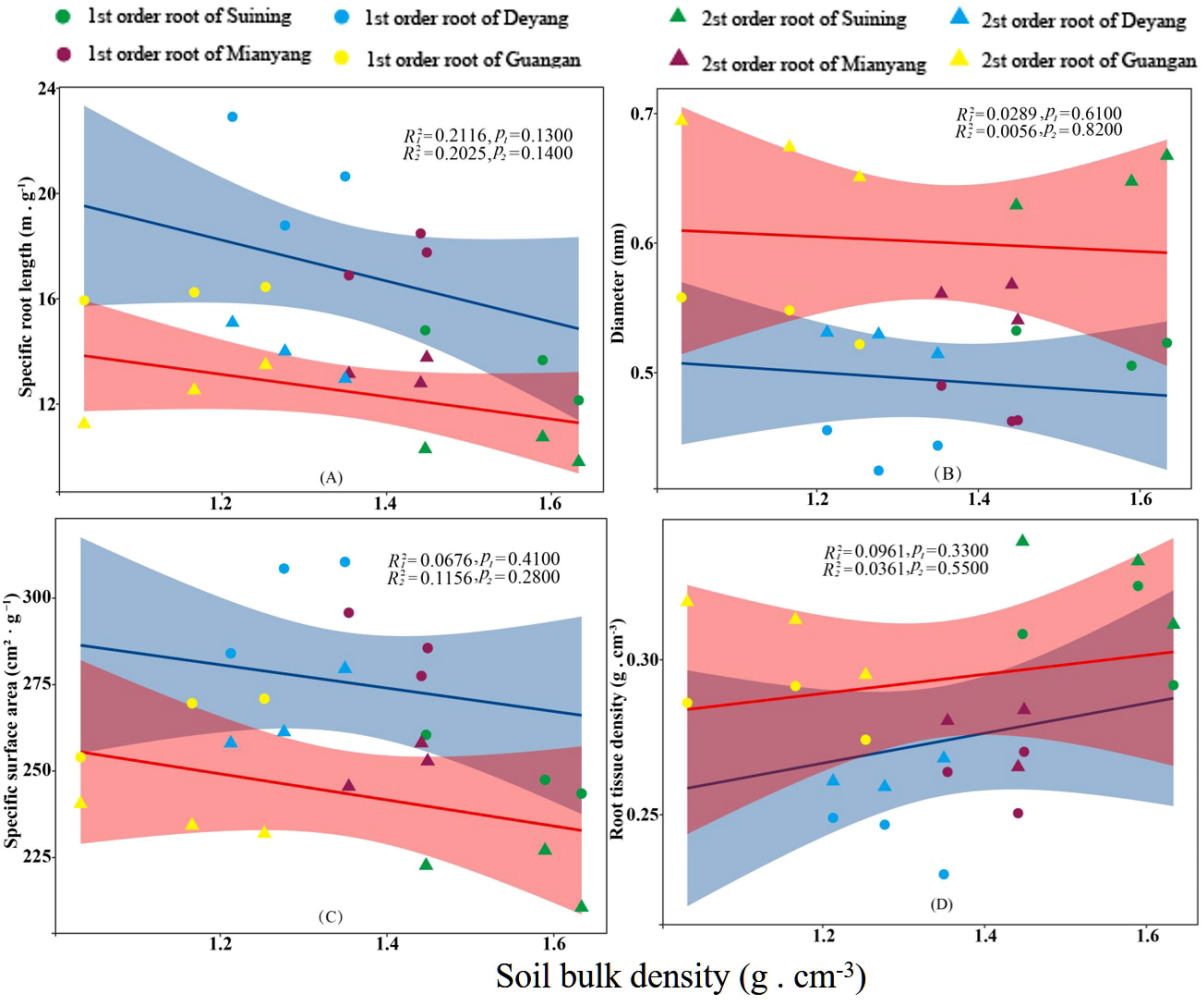
Effects of soil bulk density on specific root length, root diameter, specific root surface area, and root tissue density at four research sites. *R_1_^2^* and *R_2_^2^* represent coefficients of determination for regressions of first- and second-order fine roots * and ** indicate significance at *p* < 0.05 and *p* < 0.01, respectively. *p*_1_ and *p*_2_ are p-values indicating significance of regressions with first- and second-order fine roots, respectively. The shaded part is the confidence interval of the fitting line, regimes (n = 3 per treatment).

**Figure 9:**
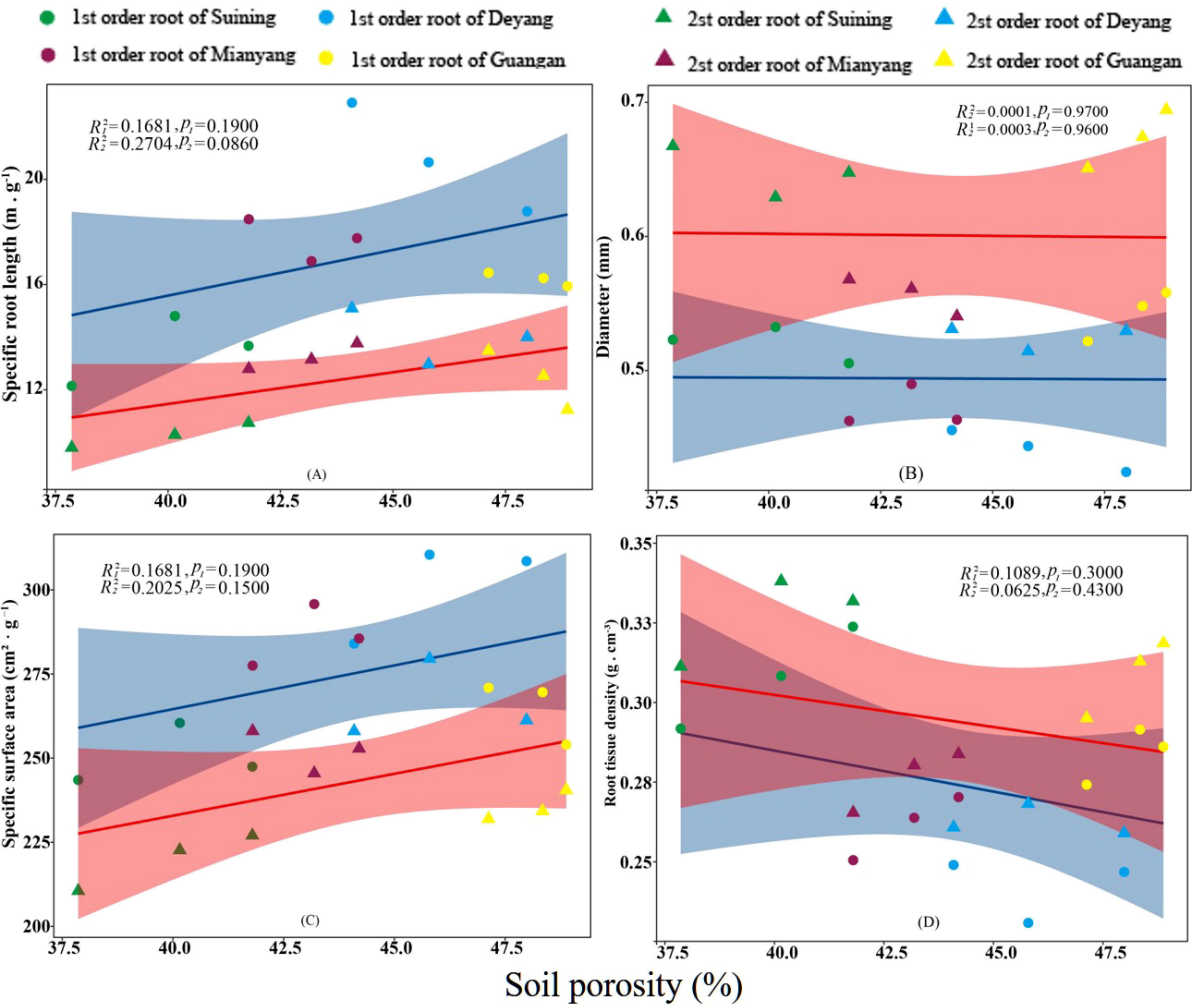
Effects of soil porosity on specific root length, root diameter, specific root surface area, and root tissue density at four research sites. *R_1_^2^* and *R_2_^2^* represent coefficients of determination for regressions of first- and second-order fine roots * and ** indicate significance at *p* < 0.05 and *p* < 0.01, respectively. *p*_1_ and *p*_2_ are p-values indicating significance of regressions with first- and second-order fine roots, respectively. The shaded part is the confidence interval of the fitting line, regimes (n = 3 per treatment).

The results showed that SRA and SRL of first- and second -order fine roots were directly proportional to TN content (**Figures 2C, A**, respectively). The TN content was negatively correlated with the RTD of first -order fine roots and D of first- and second -order fine roots (**Figures 2D, B**, respectively). The content of AN was positively correlated with SRL and SRA of the first- and second -order fine roots (**Figures 3A, C**, respectively), and negatively correlated with RTD and D (**Figures 3D, B**, respectively). OC content did not show significant changes with SRL, SRA, D and RTD of first- and second-order fine roots (**Figure 4**). AP increased, SRA increased in first- and second -order fine roots, and SRL increased in first -order roots. AP content was positively correlated with SRA and SRL of first- and second -order fine roots (**Figures 5A, C**, respectively). AP content was negatively correlated with the RTD of first-order fine roots and D of second-order fine roots (**Figures 5B, D**, respectively). SW was positively correlated with D and RTD of first- and second-order fine roots (**Figures 6B, D**, respectively); it was negatively correlated with SRL of first -order fine roots and SRA of first- and second -order fine roots (**Figures 6A, C**, respectively). ST, SBD, and SP were not correlated with SRL, SRA, D, and RTD for first- and second -order fine roots (**Figures 7**–**9**, respectively).

In different soils, SW, AN, TN, and AP were related to fine root morphology. Among those soil properties, AN, TN, and SW content were strong. Soil OC, ST, SBD, and SP were not significantly related to fine root morphology.

### Main soil environmental factors that affected the morphology of *Cupressus funebris* fine roots

**Figure 10:**
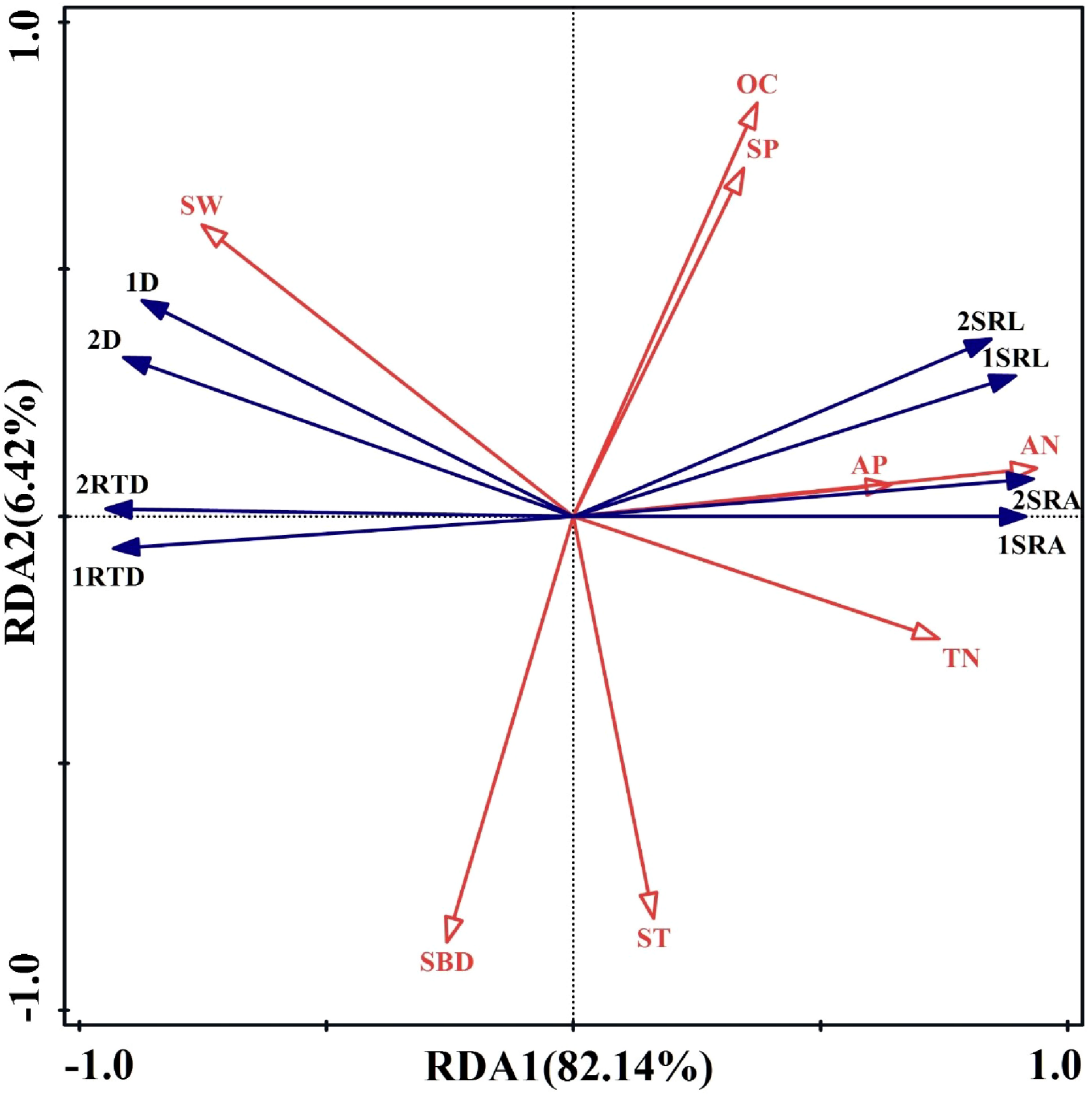
Redundancy analysis between soil environmental factors and morphological characteristics of first- and second-order fine roots. 1 and 2 represent first- and second-order roots, respectively; SRL, specific root length; SRA, specific surface area; D, diameter; RTD, root tissue density; AN, alkaline nitrogen; AP, available phosphorus; OC, organic carbon; TN, total nitrogen; ST, soil temperature; SW, soil moisture; SBD, soil bulk density; SP, soil porosity.

**Figure 11:**
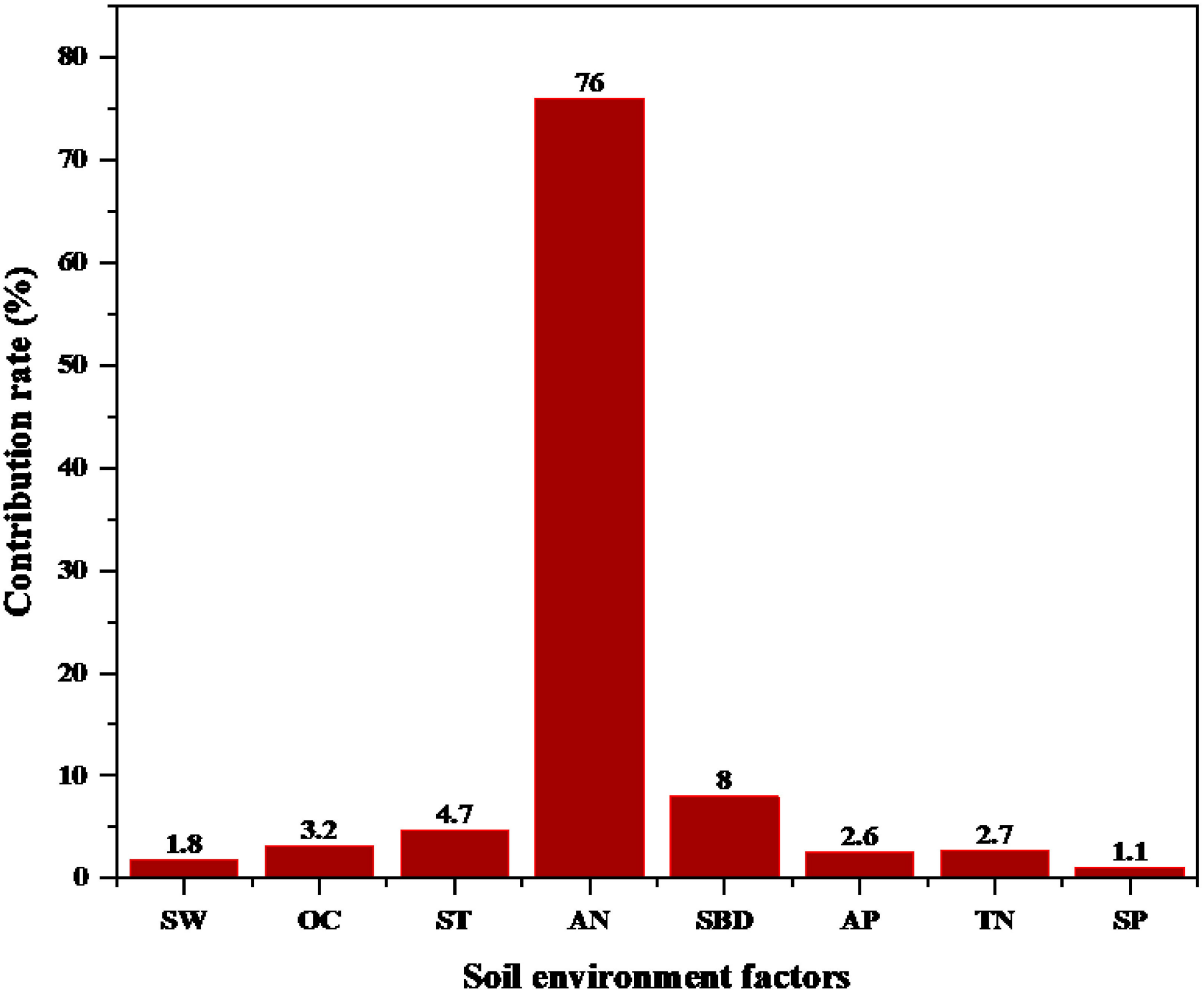
Contribution rates of soil environmental factors affecting fine root morphology. AN, alkaline nitrogen; SBD, soil bulk density; AP, available phosphorus; SW, soil moisture; OC, organic carbon; ST, soil temperature; TN, total nitrogen; SP, soil porosity.

Because of the relations between soil environmental factors and fine root morphology, RDA was used to analyze the importance of relations between fine root morphology (response variables) and soil environmental factors (explanatory variables). The first-sort axis explained 82.14% of total spatial variation, and the second-sort axis explained 6.42%, resulting in 88.56% of the total variation explained (**Figure 10**). Thus, the first two axes well reflected the relations between fine root morphology and soil environmental factors. In **Figure 10**, the angles between the SRL, SRA, AN, and TN were sharp, and the angle between the SW was obtuse for the first- and second -order fine roots. The angle between the D, RTD, and AP, AN, and TN was obtuse, and the angle between the SW was sharp for the first- and second -order fine roots. The angle between SRL, SRA, D, and RTD and SBD, ST, OC, and SP of first- and second -order fine roots was close to the right angle. This indicated that the SRL, SRA of first- and second -order fine roots were positively correlated with AP, AN, and TN and were negatively correlated with SW. However, the D and RTD of first- and second -order fine roots were positively correlated with SW and negatively correlated with AN, AP, and TN. The SRL, SRA, D, RTD, and while SBD, OC, SP, and ST of first- and second-order fine roots did not show a correlation. According to the RDA, AN was the main factor influencing fine root morphology, contributing 76% (**Figure 11**). The results indicated that the fine root morphology was also influenced by SBD, which contributed 8%. But the contribution rate was only 8%. Therefore, among the environmental indicators, soil AN had the greatest effect on fine root morphology and was the leading factor driving changes in the characteristics of fine root morphology.

## Discussion

This study investigated the fine root morphological responses of first- to fifth -order fine roots in different soil environments. The results showed that fine roots adapted to different soil environments through morphological plasticity. Fine root morphology has been demonstrated by many studies to be influenced by the soil environment (Hill et al., 2006; Addo-Danso et al., 2019; Zhang et al., 2020). However, it has been also found that fine root morphology did not change with changes in soil nutrient content (Weemstra et al., 2016b), and this might be due to the developmental conservation of fine root morphology under nutrient-rich plates (Liu et al., 2015). Under normal conditions, fine root morphology responded to different soil environments.

### Morphological changes of fine roots in different root orders

With the increase in root order, fine roots were mainly divided into absorbing roots and transporting roots (Qin et al., 2021). It was generally believed that the fourth- and fifth-roots were transportation roots (Guo et al., 2008; McCormack et al., 2015). However, the classification of absorbing roots seemed unclear. Some literature found that the morphology of first- and second -order roots changed with the soil environment (Li et al., 2017), while others found that first- to third -order fine roots changed (Gu et al., 2014; Wang et al., 2019). Our research results showed that only the first- and second -order fine roots changed with the soil environment, while the third -order fine root did not respond. According to the hypothesis of the ephemeral root model, the main physiological function of low roots (first- and second -order fine roots) was to absorb nutrients and moisture (Pregitzer et al., 2002), and they responded to the availability of soil resources through morphological plasticity (Trumbore and Gaudinski, 2003; Wang et al., 2013). And the thicker roots (third- to fifth -order fine roots) were mainly responsible for storing and transporting nutrients and supporting the aboveground parts (Bassirirad et al., 1993; Ouimet et al., 2008). Our research results supported this hypothesis. In addition, we had previously found that the action of third -order fine roots would slip (when the environment was different, the third root -order fine root function was unstable). It tended to absorb function in some environments and transport function in other environments (Li et al., 2022). It was generally accepted that fine roots differ in structure depending on the root order (Guo et al., 2008). However, studies have found that fine roots with different root orders might have similar structural functions. Such as cortical thickness (Gu et al., 2014), third -order fine roots would change from absorbing roots to transporting roots as soil depths varied (Wang et al., 2019). Consequently, we speculated that the third -order roots functions were varied due to the different structure and function among different root orders and different soil environment. And the specific reasons were needed to go a step further study.

### Relationship between fine root morphology and soil nutrients

Nitrogen was an important plant nutrient element (Shen et al., 2022), and the nitrogen cycle directly affected ecosystem productivity (Gruber and Galloway, 2008; LeBauer and Treseder, 2008; Xu and He, 2020). Nitrogen in the soil would affect fine root morphology (Brassard et al., 2009; Razaq et al., 2017), thus affecting the chemical composition of plants (Li et al., 2010; Mukta and Sreevalli, 2010). AN was a form of nitrogen in forest soil that was more critical than TN (Wallis et al., 2010). AN was an important indicator for evaluating soil nutrients and could affect nutrient cycling at the ecosystem level (Reich et al., 1997; Gong et al., 2020). This study found that the most significant environmental factor affecting the morphological changes of first- and second -order fine roots was soil AN (76%), and similar literature has been reported (Yu et al., 2017; Zhang et al., 2020). In many studies, AN significantly changed the morphological characteristics of roots (Drew, 1975; Wang et al., 2013; Chen et al., 2016). As the increase in AN content led to increase in photosynthesis and transpiration in trees (Carbone and Trumbore, 2007; Wang and Liu, 2014), the activity and capacity of plant-absorbing roots increased, leading to changes of fine root morphology. While the morphology and physiological activity of coarse roots were not affected (Adamtey et al., 2010; Bekku et al., 2011; Wang et al., 2017). AN was not easily leached from soil (Chen et al., 2016), and it could be directly absorbed by plants to affect growth (Schimel and Bennett, 2004; Li et al., 2013; Yu et al., 2020). This might mean that AN could change the morphology of fine roots by directly absorbing roots from plants. With the increase in soil nitrogen content, the respiration rate and activity of fine roots were enhanced (Wang et al., 2017; Bergmann et al., 2020), the nutrient absorption efficiency of fine roots was improved (Geng and Jin, 2022), and the transport capacity of fine roots was improved, thus driving the morphology of fine roots (Wang et al., 2018). Nitrogen in the soil changed the respiration rate of fine roots by affecting the concentration of root nitrogen (Jia et al., 2011), thus increasing the absorption and transport of ions in fine roots to maintain root growth (Lucas et al., 2011). The increase in soil nitrogen content increased the SRL and SRA of fine roots, decreased RTD (Makita et al., 2009; Noguchi et al., 2013; Geng and Jin, 2022), and changed the morphology of first- and second- order fine roots (Wang et al., 2013). AN in the soil also affected soil microbial function (Levy-Booth et al., 2014; Cong et al., 2015). For this reason, we inferred that AN might also promote root respiration by affecting the function of soil microorganisms, and the ions absorption by fine roots was affected by the increase of fine root activity. Finally, fine root morphology changed.

In many forest soil environments, phosphorus was the main limiting factor (Lang et al., 2017). Studies have found that the content of soil phosphorus increased, with the SRL and SRA of fine roots increasing and the RTD decreasing (Wurzburger and Wright, 2015; Jia et al., 2021). And this study also confirmed this conclusion. Root morphology were related to phosphatase activity (Ushio et al., 2015). Phosphatase activity in roots was positively correlated with SRL and SRA and negatively correlated with D (Ushio et al., 2015; Lugli et al., 2019). Hence, we inferred that the change in phosphorus content might stimulate the phosphatase activity of roots and promote the response of fine root morphology. However, phosphorus did not flow in the soil, and it was not readily available to plants (Aerts, 1999; Ma et al., 2020). Only a small amount of AP dissolved in soil solutions could be directly absorbed and utilized by plants (Fujita et al., 2010; Lambers, 2022). In the subtropical region of China, the content of soil phosphorus was relatively low (He et al., 2003), while the diffusion of AP in soil was relatively slow (Lewis and Quirk, 1967). Conifers might have conservative life strategies (Coomes et al., 2005), thus showing low morphological plasticity (Grassein et al., 2010). This may be the reason why AP contributes less to fine root morphology than AN. The soil was the largest repository of OC in the terrestrial ecosystem (Post et al., 1982). Plants released OC into the soil through their roots through photosynthesis, which eventually became soil organic matter (Rasse et al., 2005; Clemmensen et al., 2013; Jackson et al., 2017), and the process was usually called root deposition (Jones et al., 2004). However, root deposition occurred unidirectionally, so plants rarely used carbon in reverse (Trolldenier, 1987). Although the roots might also obtain a small amount of previously lost carbon element from the soil under the action of root exudates when OC existed in dissolved form, the roots could hardly directly control this process (Jones and Darrah, 1996). This meant that the fine root morphology was difficult to respond to the OC in the soil. This may be why OC in this study did not have a significant correlation with fine root morphology.

### Relationship between fine root morphology and soil moisture

We found that with the decrease in SW content, D and RTD decreased, while SRL and SRA increased. And the same findings have been reported in previous studies. Such as, when SW content decreased, root SRL increased and D decreased (Zhou et al., 2018). According to the theory of the optimal allocation of resources, when the moisture supply was insufficient, plants would increase the root absorption area (Hertel et al., 2013), and the root moisture absorption efficiency would increase (Dhiman et al., 2017). The decrease of fine root D and RTD and the increase of SRL and SRA indicated that fine roots adapted to a thinner and longer morphology (White and Kirkegaard, 2010; McCormack et al., 2015). It could increase the contact area between plants and SW and improve the efficiency of soil volume use (Wasson et al., 2012). Less D and more SRL could reduce the ectoplasmic barrier of root xylem moisture to increase moisture transmission (Wasson et al., 2012; Comas et al., 2013), which was conducive to fine roots using soil nutrients more effectively (Preditzer et al., 2000; Lobet et al., 2014). This might be the reason why D and RTD decreased and SRL and SRA increased when SW content decreased in our study. At the same time, some studies found that when moisture content decreased, transpiration and respiration decreased, and the biomass of fine roots decreased (Zang et al., 2014). For example, in order to reduce moisture loss caused by transpiration, plants would allocated biomass to more durable roots under drought conditions (Brunner et al., 2015). As a result, we extrapolated that when the moisture content was low, plants might also be able to adjust fine root morphology according to biomass distribution so as to optimize moisture absorption and make the plant still grow well when moisture was insufficient. When the soil is well hydrated, the fine roots increase D and RTD, and the cell walls of phloem cells were strongly lignified, which improves the efficiency of moisture transport (Blokhina et al., 2003). This experiment found that D and RTD decreased and SRL and SRA increased when SW content increased. This may be the result of fine root morphology change caused by SW distribution. However, in the RDA analysis of this study, the contribution of SW to fine root morphology was small (contribution rate of 1.8%, **Figure 9**). It could be seen that SW might contribute to fine root morphology, but it was not the main environmental factor that causes fine root morphology changes.

### Fine root morphology affected by soil physical properties

ST was related to fine root growth. It had been found that the temperature of Flakaliden coniferous forest increased the SRL of fine roots (Leppalammi-Kujansuu et al., 2013). Norwegian spruce roots were also affected by ST (Kilpeläinen et al., 2019). In warm soil, SRL and SRA of spruce absorbing roots was increased, while RTD was decreased (Parts et al., 2018). At the same time, a high-temperature environment might reduce the growth of fine root D (Lyr and Garbe, 1995; Qin et al., 2007). The root length and root surface area were not affected at 24°C but decreased seriously at 28°C. This indicated that root morphology might have some adaptability to temperature (Sefloo et al., 2021). Nevertheless, fine root morphology did not show a response to ST in our study. From this point of view, it might be that the ST did not reach the appropriate level of fine root morphology change. Furthermore, some studies believed that the response of plants to temperature would vary from species to species. The law of factor complementarity held that the temperature difference would also be compensated by nitrogen, phosphorus, potassium, and other nutrient elements (Maurel and Nacry, 2020). Accordingly, we speculated that different species or nutrient complementation might also affect our research results.

The growth of fine roots would change with SBD (He et al., 2022). Some studies had shown that when roots grew in soil with large SBD and small pores, the axial growth of cells would be limited while the tangential growth would increase (Bengough et al., 1997). The length of the root system would also increase significantly with the increase in SBD (Ola et al., 2018). In hard soils, root growth might also differ between young and mature growth stages (Burr-Hersey et al., 2017). In this study, fine root morphology was not affected by SBD but showed a certain contribution rate. Synergistic or antagonistic effects of soil physical and chemical properties might weaken the impact of soil compaction. Some studies also showed that the growth of fine roots was the result of the combined effects of SW, ST, and nutrients (Preditzer et al., 2000). As a consequence, we deduced that the results of this study might be due to the combined effect of moisture and nutrients. This might also be caused by other reasons, and we look forward to further research.

## Conclusion

We evaluated the response strategies of different root order fine root morphology to soil environment. We concluded that there were significant differences in fine root morphology between different root orders. Soil environments had significant effects on fine root morphology in different root orders. Fine roots adapted to environmental changes by changing root morphology, which was mainly reflected in the changes in first- and second -order fine root morphology. There was no significant morphological change on third- to fifth -order fine roots. In different soil environments, the contribution of soil AN to fine root morphology was particularly important, accounting for 76% of the total contribution. Soil AN promoted the efficient absorption of plant nutrients by increasing the SRL and SRA of first- and second -order fine roots and reducing the D and RTD of first- and second -order fine roots, respectively. These findings provided insights into adaptive strategies for predicting changes in fine root morphology in different soil environments. However, whether fine roots were similarly affected in other species or other environments was worthy of further study.

## Data availability statement

The raw data supporting the conclusions of this article will be made available by the authors, without undue reservation.

## Author contributions

XCW, MY and CF conceived and designed the experiments. XCW, XW, GH, YW and TL performed the experiments. XCW, HL and KZ analyzed the data. XCW, GC and WH wrote the manuscript; other authors provided editorial advice. All authors contributed to the article and approved the submitted version.

## References

[B1] Adamtey N. Cofie O. Ofosu-Budu K. G. Ofosu-Anim J. Laryea K. B. Forster D. (2010). Effect of n-enriched co-compost on transpira-tion efficiency and water-use efficiency of maize (*Zea mays l.*) under controlled irrigation. Agric. Water Manage. 97, 995–1005. doi: 10.1016/j.agwat.2010.02.004

[B2] Addo-Danso S. D. Defrenne C. E. McCormack M. L. Ostonen I. Addo-Danso A. Foli E. G.. (2019). Fine-root morphological trait variation in tropical forest ecosystems: an evidence synthesis. Plant Ecol. 221, 1–13. doi: 10.1007/s11258-019-00986-1

[B3] Addo-Danso S. D. Prescott C. E. Adu-Bredu S. Duah-Gyamfi A. Moore S. Guy R. D.. (2018). Fine-root exploitation strategies differ in tropical old growth and logged-over forests in Ghana. Biotropica. 50, 606–615. doi: 10.1111/btp.12556

[B4] Aerts R. (1999). Interspecific competition in natural plant communities: mechanisms, trade-offs and plant-soil feedbacks. J. Exp. Bot. 50, 29–37. doi: 10.1016/S0142-9612(01)00370-2

[B5] Bakker M. R. Augusto L. Achat D. L. (2006). Fine root distribution of trees and understory in mature stands of maritime pine (Pinus pinaster) on dry and humid sites. Plant Soil. 286, 37–51. doi: 10.1007/s11104-006-9024-4

[B6] Bardgett R. D. Mommer L. Vries F. T. (2014). Going underground: Root traits as drivers of ecosystem processes. Trends Ecol. Evol. 29, 692–699. doi: 10.1016/j.tree.2014.10.006 25459399

[B7] Bassirirad H. Caldwell M. M. Bilbrough C. (1993). Effects of soil temperature and nitrogen status on kinetics of 15NO_3_ ^-^uptake by roots of field-grown agropyron desertorum (*Fisch. ex link*) schult. New Phytol. 123, 485–489. doi: 10.1111/j.1469-8137.1993.tb03760.x 33874114

[B8] Bekku Y. S. Sakata T. Tanaka T. Nakano T. (2011). Midday depression of tree root respiration in relation to leaf transpiration. Ecol. Res. 26, 791–799. doi: 10.1007/s11284-011-0838-z

[B9] Bengough A. G. Mullins C. E. Wilson G. (1997). Estimating soil frictional resistance to metal probes and its relevance to the penetration of soil by roots. Eur. J. Soil Sci. 48, 603–612. doi: 10.1111/j.1365-2389.1997.tb00560.x

[B10] Bergmann J. Weigelt A. van der Plas F. Laughlin D. C. Kuyper T. W. Guerrero-Ramirez N.. (2020). The fungal collaboration gradient dominates the root economics space in plants. Sci. Advances. 6, eaba3756. doi: 10.1126/sciadv.aba3756 32937432 PMC7458448

[B11] Blokhina O. Virolainen E. Fagerstedt K. V. (2003). Antioxidants, oxidative damage and oxygen deprivation stress: a review. Ann. Bot-London. 91, 179–194. doi: 10.1093/aob/mcf118 PMC4244988 12509339

[B12] Brassard B. W. Chen H. Y. H. Bergeron Y. (2009). Influence of environmental variability on root dynamics in northern forests. Crit. Rev. Plant Sci. 28, 179–197. doi: 10.1080/07352680902776572

[B13] Brunner I. Herzog C. Dawes M. A. Arend M. Sperisen C. (2015). How tree roots respond to drought. Front. Plant Sci. 6. doi: 10.3389/fpls.2015.00547 PMC4518277 26284083

[B14] Burr-Hersey J. E. Mooney S. J. Bengough A. G. Mairhofer S. Ritz K. (2017). Developmental morphology of cover crop species exhibit contrasting behaviour to changes in soil bulk density, revealed by X-ray computed tomography. PloS One 12, e0181872. doi: 10.1371/journal.pone.0181872 28753645 PMC5533331

[B15] Carbone M. S. Trumbore S. E. (2007). Contribution of new photosynthetic assimilates to respiration by perennial grasses and shrubs: Residence times and allocation patterns. New Phytol. 176, 124–135. doi: 10.1111/j.1469-8137.2007.02153.x 17803644

[B16] Chapin F. S. (1983). Adaptation of selected trees and grasses to low availability of phosphorus. Plant Soil. 72, 283–287. doi: 10.1007/bf02181967

[B17] Chen Y. F. Qiang J. Y. Ouyang A. L. (2021). Relationship between pH value and physical and chemical properties of tobacco-growing soil. Agric. Biotechnol. 10, 99–102+106. doi: 10.19759/j.cnki.2164-4993.2021.02.023

[B18] Chen W. Koide R. T. Adams T. S. DeForest J. L. Cheng L. Eissenstat D. M. (2016). Root morphology and mycorrhizal symbioses together shape nutrient foraging strategies of temperate trees. Proc Natl Acad Sci USA. 113, 8741–8746. doi: 10.1073/pnas.1601006113 27432986 PMC4978252

[B19] Clark L. J. Whalley W. R. Barraclough P. B. (2003). How do roots penetrate strong soil? Plant Soil. 255, 93–104. doi: 10.1007/978-94-017-2923-9_10

[B20] Clemmensen K. E. Bahr A. Ovaskainen O. Dahlberg A. Ekblad A. Wallander H.. (2013). Roots and associated fungi drive long-term carbon sequestration in Boreal forest. Science. 339, 1615–1618. doi: 10.1126/science.1231926 23539604

[B21] Comas L. Becker S. Cruz V. M. Byrne P. F. Dierig D. A. (2013). Root traits contributing to plant productivity under drought. Front. Plant Sci. 4. doi: 10.3389/fpls.2013.00442 PMC3817922 24204374

[B22] Cong J. Liu X. Lu H. Xu H. Li Y. Deng Y.. (2015). Available nitrogen is the key factor influencing soil microbial functional gene diversity in tropical rainforest. BMC Microbiol. 15, 167. doi: 10.1186/s12866-015-0491-8 26289044 PMC4546036

[B23] Coomes D. A. Allen R. B. Bentley W. A. Burrows L. E. Canham C. D. Fagan L.. (2005). The hare, the tortoise and the crocodile: the ecology of angiosperm dominance, conifer persistence and fern filtering. J. Ecol. 93, 918–935. doi: 10.1111/j.13652745.2005.01012.x

[B24] De Andrade L. I. F. Linhares P. C. A. da Fonseca T. M. da Silva A. A. dos Santos J. P. Pereira M. P.. (2022). Photosynthetic efficiency and root plasticity promote drought tolerance in coffee genotypes. Acta Physiol. Plant 44, 109. doi: 10.1007/s11738-022-03434-2

[B25] Defrenne C. E. McCormack M. L. Roach W. J. Addo-Danso S. D. Simard. S. W. (2019). Intraspecific fine-root trait-environment relationships across interior douglas-fir forests of western canada. Plants. 8, 199. doi: 10.3390/plants8070199 31262042 PMC6681360

[B26] Dhiman I. Bilheux H. Decarlo K. Painter S. L. Santodonato L. Warren J. M. (2017). Quantifying root water extraction after drought recovery using sub-mm in situ empirical data. Plant Soil. 424, 73–89. doi: 10.1007/s11104-017-3408-5

[B27] Drew M. C. (1975). Comparison of the effects of a localised supply of phosphate, nitrate, ammonium and potassium on the growth of the seminal root system, and the shoot, in barley. New Phytol. 75, 479–490. doi: 10.1111/j.1469-8137.1975.tb01409.x

[B28] Eissenstat D. M. Wells C. E. Yanai R. D. Whitbeck J. L. (2000). Building roots in a changing environment: implications for root longevity. New Phytol. 147, 33–42. doi: 10.1046/j.1469-8137.2000.00686.x

[B29] Fan P. P. Jiang Y. X. (2010). Nitrogen dynamics differed among the first six root branch orders of *Fraxinus mandshurica* and *Larix gmelinii* during short-term decomposition. J. Plant Res. 123, 433–438. doi: 10.1007/s10265-009-0303-z 20082111

[B30] Finér L. Ohashi M. Noguchi K. Hirano Y. (2011). Factors causing variation in fine root biomass in forest ecosystems. For. Ecol. Manage. 261, 0–277. doi: 10.1016/j.foreco.2010.10.016

[B31] Fransen B. de Kroon H. (2001). Long-term disadvantages of selective root placement: root proliferation and shoot biomass of two perennial grass species in a 2-year experiment. J. Ecol. 89, 711–722. doi: 10.1046/j.0022-0477.2001.00589.x

[B32] Freschet G. T. Roumet C. Comas L. H. Weemstra M. Bengough A. G. Rewald B.. (2021). Root traits as drivers of plant and ecosystem functioning: current understanding, pitfalls and future research needs. New Phytol. 232, 1123–1158. doi: 10.1111/NPH.17072 33159479

[B33] Fujita Y. Robroek B. J. M. De Ruiter P. C. Heil G. W. Wassen M. J. (2010). Increased n affects p uptake of eight grassland species: the role of root surface phosphatase activity. Oikos. 119, 1665–1673. doi: 10.1111/j.1600-0706.2010.18427.x

[B34] Geng P. Jin G. (2022). Fine root morphology and chemical responses to n addition depend on root function and soil depth in a Korean pine plantation in northeast China. For. Ecol. Manage. 520, 120407. doi: 10.1016/j.foreco.2022.120407

[B35] Gong S. Zhang T. Guo J. (2020). Warming and nitrogen deposition accelerate soil phosphorus cycling in a temperate meadow ecosystem. Soil Res. 58, 109–115. doi: 10.1071/SR19114

[B36] Grassein F. Till-Bottraud I. Lavorel S. (2010). Plant resource-use strategies: the importance of phenotypic plasticity in response to a productivity gradient for two subalpine species. Ann. Bot. 106, 637–645. doi: 10.1093/aob/mcq154 20682576 PMC2944977

[B37] Gruber N. Galloway J. N. (2008). An earth-system perspective of the global nitrogen cycle. Nature. 451, 293–296. doi: 10.1038/nature06592 18202647

[B38] Guo D. Xia M. Wei X. Chang W. Liu Y. Wang Z. (2008). Anatomical traits associated with absorption and mycorrhizal colonization are linked to root branch order in twenty-three Chinese temperate tree species. New Phytol. 180, 673–683. doi: 10.1111/j.1469-8137.2008.02573.x 18657210

[B39] Gu J. Xu Y. Dong X. Wang H. Wang Z. (2014). Root diameter variations explained by anatomy and phylogeny of 50 tropical and temperate tree species. Tree Physiol. 34, 415–425. doi: 10.1093/treephysu019 24695727

[B40] He Y. Liao H. Yan X. (2003). Localized supply of phosphorus induces root morphological and architectural changes of rice in split and stratified soil cultures. Plant Soil. 248, 247–256. doi: 10.1023/A:1022351203545

[B41] Hertel D. Strecker T. Müller-Haubold H. Leuschner C. Guo D. (2013). Fine root biomass and dynamics in beech forests across a precipitation gradient–is optimal resource partitioning theory applicable to water-limited mature trees? J. Ecol. 101, 1183–1200. doi: 10.1111/1365-2745.12124

[B42] He Y. Wu Z. Zhao T. Yang H. Ali W. Chen J. (2022). Different plant species exhibit contrasting root traits and penetration to variation in soil bulk density of clayey red soil. Agron. J. 114, 867–877. doi: 10.1002/agj2.20972

[B43] Hill J. O. Simpson R. J. Moore A. D. Chapman D. F. (2006). Morphology and response of roots of pasture species to phosphorus and nitrogen nutrition. Plant Soil. 286, 7–19. doi: 10.1007/s11104-006-0014-3

[B44] Hodge A. Berta G. Doussan C. Merchan F. Crespi M. (2009). Plant root growth, architecture and function. Plant Soil. 321, 153–187. doi: 10.1007/s11104-009-9929-9

[B45] Iversen C. M. McCormack M. L. Powell A. S. Blackwood C. B. Freschet G. T. Freschet J.. (2017). A global fine-root ecology database to address below-ground challenges in plant ecology. New Phytol. 215, 15–26. doi: 10.1111/nph.14486 28245064

[B46] Jackson R. B. Lajtha K. Crow S. E. Hugelius G. Kramer M. G. Piñeiro G. (2017). The ecology of soil carbon: Pools, vulnerabilities, and biotic and abiotic controls. Annu. Rev. Ecol. Evol. S. 48, 419–445. doi: 10.1146/annurev-ecolsys-112414-054234

[B47] Jia S. Li X. Sun W. Wang Q. Liu H. Zhou C.. (2021). Fine root traits of *Pinus koraiensis* varied with soil cation exchange capacity in natural forests. Land. 10, 363. doi: 10.3390/land10040363

[B48] Jia S. Wang Z. Li X. Zhang X. Mclaughlin N. B. (2011). Effect of nitrogen fertilizer, root branch order and temperature on respiration and tissue n concentration of fine roots in *Larix gmelinii* and *Fraxinus mandshurica* . Tree Physiol. 31, 718–726. doi: 10.1093/treephys/tpr057 21849591

[B49] Jones D. L. Darrah P. R. (1996). Re-sorption of organic compounds by roots of zea mays l. and its consequences in the rhizosphere. III characteristics of sugar influx and efflux. Plant Soil. 178, 153–160. doi: 10.1007/BF00011173

[B50] Jones D. L. Hodge A. Kuzyakov Y. (2004). Plant and mycorrhizal regulation of rhizodeposition. New Phytol. 163, 459–480. doi: 10.1111/j.1469-8137.2004.01130.x 33873745

[B51] Keeney D. R. Bremner J. M. (1966). Comparison and evaluation of laboratory methods of obtaining an index of soil nitrogen availability. Agron. J. 58, 498–503. doi: 10.2134/agronj1966.00021962005800050013x

[B52] Kengdo S. W. Peršoh D. Schindlbacher A. Heinzle J. Tian Y. Wanek W.. (2022). Long-term soil warming alters fine root dynamics and morphology, and their ectomycorrhizal fungal community in a temperate forest soil. Global Change Biol. 28, 3441–3458. doi: 10.1111/gcb.16155 35253326

[B53] Kilpeläinen J. Domisch T. Lehto T. Finér L. Aphalo P. J. Leinonen I.. (2019). Root and shoot phenology and root longevity of Norway spruce saplings grown at different soil temperatures. Can. J. For. Res. 49, 1441–1452. doi: 10.1139/cjfr-2019-0190

[B54] Lambers H. (2022). Phosphorus acquisition and utilization in plants. Ann. Rev. Plant Biol. 73, 11–126. doi: 10.1146/annurev-arplant-102720-125738 34910587

[B55] Lang F. Krüger J. Amelung W. Willbold S. Frossard E. Bünemann E. K.. (2017). Soil phosphorus supply controls p nutrition strategies of beech forest ecosystems in central Europe. Biogeochemistry. 136, 5–29. doi: 10.1007/s10533-017-0375-0

[B56] LeBauer D. S. Treseder K. K. (2008). Nitrogen limitation of net primary productivity in terrestrial ecosystems is globally distributed. Ecology. 89, 371–379. doi: 10.1890/06-2057.1 18409427

[B57] Lee C. Wang Y. J. (2017). Psychrometer based on a contactless infrared thermometer with a predictive model for water evaporation. Biosyst. Eng. 160, 84–94. doi: 10.1016/j.biosystemseng.2017.05.010

[B58] Leppalammi-Kujansuu J. Ostonen I. Strömgren M. Nilsson L. O. Kleja D. B. Sah S. P.. (2013). Effects of long-term temperature and nutrient manipulation on Norway spruce fine roots and mycelia production. Plant Soil. 366, 287–303. doi: 10.1007/s11104-012-1431-0

[B59] Leuschner C. Hertel D. Schmid I. Oliver K. Muhs A. Hölscher D. (2004). Stand fine root biomass and fine root morphology in old-growth beech forests as a function of precipitation and soil fertility. Plant Soil. 258, 43–56. doi: 10.1023/B:PLSO.0000016508.20173.80

[B60] Levy-Booth D. J. Prescott C. E. Grayston S. J. (2014). Microbial functional genes involved in nitrogen fixation, nitrification and denitrification in forest ecosystems. Soil Biol. Biochem. 75, 11–25. doi: 10.1016/j.soilbio.2014.03.021

[B61] Lewis D. G. Quirk J. P. (1967). Phosphate diffusion in soil and uptake by plants. II. phosphate uptake by wheat plants. Plant Soil. 26, 119–128. doi: 10.1007/BF01978678

[B62] Liese R. Alings K. Meier I. C. (2017). Root branching is a leading root trait of the plant economics spectrum in temperate trees. Front. Plant Sci. 8. doi: 10.3389/fpls.2017.00315 PMC5340746 28337213

[B63] Li A. Guo D. Wang Z. Liu H. (2010). Nitrogen and phosphorus allocation in leaves, twigs, and fine roots across 49 temperate, subtropical and tropical tree species: A hierarchical pattern. Funct. Ecol. 24, 224–232. doi: 10.1111/j.1365-2435.2009.01603.x

[B64] Li D. Nan H. Liang J. Cheng X. Zhao C. Yin H.. (2017). Responses of nutrient capture and fine root morphology of subalpine coniferous tree *Picea asperata* to nutrient heterogeneity and competition. PloS One 12, e0187496. doi: 10.1371/journal.pone.0187496 29095947 PMC5667764

[B65] Li T. Ren J. He W. Wang Y. Wen X. Wang X.. (2022). Anatomical structure interpretation of the effect of soil environment on fine root function. Front. Plant Sci. 13. doi: 10.3389/fpls.2022.993127 PMC9470114 36110353

[B66] Liu B. Li H. Zhu B. Koide R. T. Eissenstat D. M. Guo D. (2015). Complementarity in nutrient foraging strategies of absorptive fine roots and arbuscular mycorrhizal fungi across 14 coexisting subtropical tree species. New Phytol. 208, 125–136. doi: 10.1111/nph.13434 25925733

[B67] Li W. Yang G. Chen H. Tian J. Zhang Y. Zhu Q.. (2013). Soil available nitrogen, dissolved organic carbon and microbial biomass content along altitudinal gradient of the eastern slope of *Gongga mountain* . Acta Ecologica Sinica. 33, 266–271. doi: 10.1016/j.chnaes.2013.07.006

[B68] Lobet G. Couvreur V. Meunier F. Javaux M. Draye X. (2014). Plant water uptake in drying soils. Plant Physiol. 164, 1619–1627. doi: 10.1104/pp.113.233486 24515834 PMC3982728

[B69] Lõhmus K. Truu M. Truu J. Ostonen I. Kaar E. Vares A.. (2006). Functional diversity of culturable bacterial communities in the rhizosphere in relation to fine-root and soil parameters in alder stands on forest, abandoned agricultural, and oil-shale mining areas. Plant Soil. 283, 1–10. doi: 10.1007/s11104-005-2509-8

[B70] Lucas R. W. Klaminder J. Futter M. N. Bishop K. H. Egnell G. Laudon H.. (2011). A meta-analysis of the effects of nitrogen additions on base cations: Implications for plants, soils, and streams. For. Ecol. Manage. 262, 95–104. doi: 10.1016/j.foreco.2011.03.018

[B71] Lugli L. F. Andersen K. M. Aragão L. E. O. C. Cordeiro A. L. Cunha H. F. V. Fuchslueger L.. (2019). Multiple phosphorus acquisition strategies adopted by fine roots in low-fertility soils in central Amazonia. Plant Soil. 450, 49–63. doi: 10.1007/s11104-019-03963-9

[B72] Lyr H. Garbe V. (1995). Influence of root temperature on growth of *Pinus sylvestris*, fagus sylvatica, *Tilia cordata* and *Quercus robur* . Trees. 9, 220–223. doi: 10.1007/BF00195276

[B73] Ma Q. Chen L. Du M. Zhang Y. Zhang Y. (2020). Localized and moderate phosphorus application improves plant growth and phosphorus accumulation in Rosa multiflora *Thunb. ex murr. via* efficient root system development. Forests. 11, 570. doi: 10.3390/f11050570

[B74] Makita N. Hirano Y. Dannoura M. Kominami Y. Mizoguchi T. Ishii H.. (2009). Fine root morphological traits determine variaition in root respiration of quercus serrata. Tree Physiol. 29, 579–585. doi: 10.1093/treephys/tpn050 19203981

[B75] Maurel C. Nacry P. (2020). Root architecture and hydraulics converge for acclimation to changing water availability. Nat. Plants. 6, 744–749. doi: 10.1038/s41477-020-0684-5 32601421

[B76] McCormack M. L. Dickie I. A. Eissenstat D. M. Fahey T. J. Fernandez C. W. Guo D.. (2015). Redefining fine roots improves understanding of below-ground contributions to terrestrial biosphere processes. New Phytol. 207, 505–518. doi: 10.1111/NPH.13363 25756288

[B77] McCormack M. L. Kaproth M. A. Cavender-Bares J. Carlson E. Hipp A. L. Han Y.. (2020). Climate and phylogenetic history structure morphological and architectural trait variation among fine-root orders. New Phytol. 228, 1824–1834. doi: 10.1111/nph.16804 32654151

[B78] Mei L. Gu J. Zhang Z. Wang Z. (2010). Responses of fine root mass, length, production and turnover to soil nitrogen fertilization in *Larix gmelinii* and *Fraxinus mandshurica* forests in northeastern China. J. For. Res-Jpn. 15, 194–201. doi: 10.1007/s10310-009-0176-y

[B79] Montagnoli A. (2018). Sustainable restoration of Mediterranean forests: analysis and perspective within the context of bio-based economy development under global changes. Plant Biosyst. 152, 501–501. doi: 10.1080/11263504.2018.1435588

[B80] Mukta N. Sreevalli Y. (2010). Propagation techniques, evaluation and improvement of the biodiesel plant, *Pongamia pinnata* (L.) Pierre-a review. Ind. Crop Prod. 31, 1–12. doi: 10.1016/j.indcrop.2009.09.004

[B81] Nikolova P. S. Bauerle T. L. Häberle K. H. Blaschke H. Brunner I. Matyssek R. (2020). Fine-root traits reveal contrasting ecological strategies in European beech and Norway spruce during extreme drought. Front. Plant Sci. 11. doi: 10.3389/fpls.2020.01211 PMC7438540 32903505

[B82] Noguchi K. Nagakura J. Kaneko S. (2013). Biomass and morphology of fine roots of sugi (*Cryptomeria japonica*) after 3 years of nitrogen fertilization. Front. Plant Sci. 4. doi: 10.3389/fpls.2013.00347 PMC3760069 24027575

[B83] Ola A. Schmidt S. Lovelock C. E. (2018). The effect of heterogeneous soil bulk density on root growth of field-grown mangrove species. Plant Soil. 432, 91–105. doi: 10.1007/s11104-018-3784-5

[B84] Ostonen I. Lõhmus K. Helmisaari H. S. Truu J. Meel S. (2007). Fine root morphological adaptations in scots pine, Norway spruce and silver birch along a latitudinal gradient in boreal forests. Tree Physiol. 27, 1627–1634. doi: 10.1093/treephys/27.11.1627 17669752

[B85] Ouimet R. Camiré C. Brazeau M. Moore J. D. (2008). Estimation of coarse root biomass and nutrient content for sugar maple, jack pine, and black spruce using stem diameter at breast height. Can. J. For. Res. 38, 92–100. doi: 10.1139/X07-134

[B86] Parts K. Tedersoo L. Schindlbacher A. Sigurdsson B. D. Leblans N. I. W. Oddsdóttir E. S.. (2018). Acclimation of fine root systems to soil warming: Comparison of an experimental setup and a natural soil temperature gradient. Ecosystems. 22, 457–472. doi: 10.1007/s10021-018-0280-y

[B87] Pérez-Ramos I. M. Roumet C. Cruz P. Blanchard A. Autran P. Garnier E. (2012). Evidence for a plant community economics spectrum' driven by nutrient and water limitations in a Mediterranean rangeland of southern France. J. Ecol. 100, 1315–1327. doi: 10.1111/1365-2745.12000

[B88] Post W. M. Emanuel W. R. Zinke P. J. Stangenberger A. G. (1982). Soil carbon pools and world life zones. Nature. 298, 156–159. doi: 10.1038/298156a0

[B89] Preditzer K. S. King J. S. Burton A. J. Brown S. E. (2000). Responses of tree fine roots to temperature. New Phytol. 147, 105–115. doi: 10.1046/J.1469-8137.2000.00689.X

[B90] Pregitzer K. S. Deforest J. L. Burton A. J. Allen M. F. Ruess R. W. Hendrick R. L.. (2002). Fine root architecture of nine north american trees. Ecol. Monogr. 72, 293–309. doi: 10.1890/0012-9615(2002)072[0293:FRAONN]2.0.CO;2

[B91] Qin Y. Gao C. Jin G. Liu Z. Shi B. (2021). Latitude patterns in fine root morphological and anatomical traits across root orders of *Pinus koraiensis* . Scand. J. For. Res. 36, 539–549. doi: 10.1080/02827581.2021.1981430

[B92] Qin L. He J. Lee S. K. Dodd I. C. (2007). An assessment of the role of ethylene in mediating lettuce (Lactuca sativa) root growth at high temperatures. J. Exp. Bot. 58, 3017–3024. doi: 10.1093/jxb/erm156 17728295

[B93] Rasse D. P. Rumpel C. Dignac M. F. (2005). Is soil carbon mostly root carbon? mechanisms for a specific stabilisation. Plant Soil. 269, 341–356. doi: 10.1007/s11104-004-0907-y

[B94] Razaq M. Zhang P. Shen H. Salahuddin (2017). Influence of nitrogen and phosphorous on the growth and root morphology of acer mono. PloS One 12, e0171321. doi: 10.1371/journal.pone.0171321 28234921 PMC5325205

[B95] Reich P. B. (2014). The world-wide ‘fast–slow’ plant economics spectrum: a traits manifesto. J. Ecol. 102, 275–301. doi: 10.1111/1365-2745.12211

[B96] Reich P. B. Grigal D. F. Aber J. D. Gower S. T. (1997). Nitrogen mineralization and productivity in 50 hardwood and conifer stands on diverse soils. Ecology. 78, 335–347. doi: 10.1890/0012-9658(1997)078[0335:NMAPIH]2.0.CO;2

[B97] Roumet C. Birouste M. Picon-Cochard C. Ghestem M. Osman N. Vrignon-Brenas S.. (2016). Root structure-function relationships in 74 species: evidence of a root economics spectrum related to carbon economy. New Phytol. 210, 815–826. doi: 10.1111/nph.13828 26765311

[B98] Sato J. H. Figueiredo C. C. Marchão R. L. Madari B. E. Celino L. E. Mendes. D.. (2014). Methods of soil organic carbon determination in Brazilian savannah soils. Sci. Agr. 71, 302–308. doi: 10.1590/0103-9016-2013-0306

[B99] Schimel J. P. Bennett J. (2004). Nitrogen mineralization: challenges of a changing paradigm. Ecology. 85, 591–602. doi: 10.1890/03-8002

[B100] Schmugge T. J. Jackson T. J. McKim H. L. (1980). Survey of methods for soil moisture determination. Water Resour Res. 16, 961–979. doi: 10.1029/WR016i006p00961

[B101] Sefloo G. N. Steinkellner S. Hage-Ahmed K. (2021). The bioprotective effect of root endophytic serendipita herbamans against fusarium wilt in tomato and its impact on root traits are determined by temperature. Rhizosphere. 20, 100453. doi: 10.1016/j.rhisph.2021.100453

[B102] Shen H. Dong S. DiTommaso A. Xiao J. Lu W. Zhi Y. (2022). Nitrogen deposition shifts grassland communities through directly increasing dominance of graminoids: a 3-year case study from the qinghai-Tibetan plateau. Front. Plant Sci. 13. doi: 10.3389/fpls.2022.811970 PMC8934429 35317015

[B103] Suseela V. Tharayil N. Orr G. Hu D. (2020). Chemical plasticity in the fine root construct of quercus spp. varies with root order and drought. New Phytol. 288, 1835–1851. doi: 10.1111/nph.16841 32750158

[B104] Tan L. Fan R. Sun H. Guo S. (2021). Root foraging of birch and larch in heterogeneous soil nutrient patches under water deficit. PloS One 16, e0255848. doi: 10.1371/journal.pone.0255848 34375353 PMC8354452

[B105] Trikilidou E. Samiotis G. Tsikritzis L. Amanatidou E. (2020). Performance of semi-Micro-Kjeldahl nitrogen method – uncertainty and nitrate interference. Int. J. Environ. Anch. 1–11. doi: 10.1080/03067319.2020.1807967 (Early Access)

[B106] Trolldenier G. (1987). Curl, E.A. and B. Truelove: The Rhizosphere. (Advanced Series in Agricultural Sciences, Vol. 15) Springer-Verlag, Berlin-Heidelberg-New York-Tokyo 1986. 288 p, 57 figs., Hardcover DM 228.00, ISBN 3–540–15803–0. Z. für Pflanzenernährung und Bodenkunde. 150, 124–125. doi: 10.1002/jpln.19871500214

[B107] Trumbore S. E. Gaudinski J. B. (2003). The secret lives of roots. Science. 302, 1344–1345. doi: 10.1126/science.10918 14631025

[B108] Ugawa S. Kuninaka W. Hayata K. Maruta N. Ohashi S. Kubota V. R.. (2022). Relationship between root tip morphology and growth conditions across macaranga and shorea species in a tropical lowland forest of *Peninsula malaysia* . Plant Soil. 1–19. doi: 10.1007/s11104-022-05665-1 (Early Access)36211803

[B109] Ushio M. Fujiki Y. Hidaka A. Kitayama K. (2015). Linkage of root physiology and morphology as an adaptation to soil phosphorus impoverishment in tropical montane forests. Funct. Ecol. 29, 1235–1245. doi: 10.1111/1365-2435.12424

[B110] Valverde-Barrantes O. J. Raich J. W. Russell A. E. (2007). Fine-root mass, growth and nitrogen content for six tropical tree species. Plant Soil. 290, 357–370. doi: 10.1007/s11104-006-9168-2

[B111] Verburg P. S. J. Young A. C. Stevenson B. A. Glanzmann I. Arnone J. A. Marion G. M.. (2013). Do increased summer precipitation and n deposition alter fine root dynamics in a Mojave desert ecosystem? Glob Chang Biol. 19, 948–956. doi: 10.1111/gcb.12082 23504850

[B112] Wallis I. R. Nicolle D. Foley W. J. (2010). Available and not total nitrogen in leaves explains key chemical differences between the eucalypt subgenera. For. Ecol. Manage. 260, 814–821. doi: 10.1016/j.foreco.2010.05.040

[B113] Wang C. Brunner I. Wang J. Guo W. Geng Z. Yang X.. (2022). The right-skewed distribution of fine-root size in three temperate forests in northeastern China. Front. Plant Sci. 12. doi: 10.3389/fpls.2021.772463 PMC8777189 35069627

[B114] Wang G. Fahey T. J. Xue S. Liu F. (2013). Root morphology and architecture respond to n addition in *Pinus tabuliformis*, west China. Oecologia. 171, 583–590. doi: 10.1007/s00442-012-2441-6 22948279

[B115] Wang Y. Chen S. He W. Ren J. Wen X. Wang Y.. (2021). Shrub diversity and niche characteristics in the initial stage of reconstruction of low-efficiency cupressus funebris stands. Forests 12, 1492. doi: 10.3390/f12111492

[B116] Wang G. Liu F. (2014). Carbon allocation of Chinese pine seedlings along a nitrogen addition gradient. For Ecol. Manage. 334, 114–121. doi: 10.1016/j.foreco.2014.09.004

[B117] Wang G. Liu F. Xue S. (2017). Nitrogen addition enhanced water uptake by affecting fine root morphology and coarse root anatomy of Chinese pine seedlings. Plant Soil. 418, 177–189. doi: 10.1007/s11104-017-3283-0

[B118] Wang H. Wang Z. Dong X. (2019). Anatomical structures of fine roots of 91 vascular plant species from four groups in a temperate forest in northeast China. PloS One 14, e0215126. doi: 10.1371/journal.pone.0215126 31042717 PMC6494041

[B119] Wang W. Wang Y. Hoch G. Wang Z. Gu J. (2018). Linkage of root morphology to anatomy with increasing nitrogen availability in six temperate tree species. Plant Soil. 425, 189–200. doi: 10.1007/s11104-018-3563-3

[B120] Wasson A. P. Richards R. A. Chatrath R. Misra S. C. Prasad S. V. S. Rebetzke G. J.. (2012). Traits and selection strategies to improve root systems and water uptake in water-limited wheat crops. J. Exp. Bot. 63, 3485–3498. doi: 10.1093/jxb/ers111 22553286

[B121] Weemstra M. Mommer L. Visser E. J. Ruijven J. V. Kuyper T. W. Mohren G. M. J.. (2016a). Towards a multidimensional root trait framework: a tree root review. New Phytol. 211, 1159–1169. doi: 10.1111/nph.14003 27174359

[B122] Weemstra M. Sterck F. J. Visser E. J. Kuyper T. W. Goudzwaard L. Mommer L. (2016b). Fine-root trait plasticity of beech (*Fagus sylvatica*) and spruce (*Picea abies*) forests on two contrasting soils. Plant Soil. 415, 175–188. doi: 10.1007/s11104-016-3148-y

[B123] Wells C. E. Eissenstat D. M. (2001). Marked differences in survivorship among apple roots of different differences. Ecology. 82, 882–892. doi: 10.1890/0012-9658(2001)082[0882:MDISAA]2.0.CO;2

[B124] White R. G. Kirkegaard J. A. (2010). The distribution and abundance of wheat roots in a dense, structured subsoil–implications for water uptake. Plant Cell Environment. 33, 133–148. doi: 10.1111/j.1365-3040.2009.02059.x 19895403

[B125] Wurzburger N. Wright S. J. (2015). Fine-root responses to fertilization reveal multiple nutrient limitation in a lowland tropical forest. Ecology 96 (8), 2137–2146. doi: 10.1890/14-1362.1 26405739

[B126] Xiao H. Sheng M. Wang L. Guo C. Zhang S. (2022). Effects of short-term n addition on fine root morphological features and nutrient stoichiometric characteristics of *Zanthoxylum bungeanum* and medicago sativa seedlings in southwest China karst area. J. Soil Sci. Plant Nutt. 22, 1805–1817. doi: 10.1007/s42729-022-00773-4

[B127] Xu L. He N. (2020). Nitrogen storage and allocation in china's forest ecosystems. Sci. China-Earth Sci. 63, 1475–1484. doi: 10.1007/s11430-019-9600-5

[B128] Yang Z. Zhou B. Ge X. Cao Y. Brunner I. Shi J.. (2021). Species-specific responses of root morphology of three co-existing tree species to nutrient patches reflect their root foraging strategies. Front. Plant Sci. 11. doi: 10.3389/fpls.2020.618222 PMC7868422 33569072

[B129] Yan X. L. Jia L. Dai T. (2019). Fine root morphology and growth in response to nitrogen addition through drip fertigation in a *Populus× euramericana* “Guariento” plantation over multiple years. Ann. For. Sci. 76, 1–12. doi: 10.1007/s13595-019-0798-y

[B130] Yu Z. Wang Z. Zhang Y. Wang Y. Liu Z. (2020). Biocontrol and growth-promoting effect of trichoderma asperellum TaspHu1 isolate from juglans mandshurica rhizosphere soil. Microbiol. Res. 242, 126596. doi: 10.1016/j.micres.2020.126596 33007636

[B131] Yu R. Xia S. Liu C. Zhang Z. Shi G. (2017). Variations in root morphology among 18 herbaceous species and their relationship with cadmium accumulation. Environ. Sci. pollut. R. 24, 4731–4740. doi: 10.1007/s11356-016-8210-z 27981477

[B132] Zadworny M. McCormack M. L. Żytkowiak R. Karolewski P. Mucha J. Oleksyn J. (2017). Patterns of structural and defense investments in fine roots of scots pine (*Pinus sylvestris l.*) across a strong temperature and latitudinal gradient in Europe. Global Change Biol. 23, 1218–1231. doi: 10.1111/gcb.13514 27670838

[B133] Zang U. Goisser M. Häberle K. H. Matyssek R. Matzner E. Borken W. (2014). Effects of drought stress on photosynthesis, rhizosphere respiration, and fine-root characteristics of beech saplings: a rhizotron field study. J. Plant Nutr. Soil Sci. 177, 168–177. doi: 10.1002/jpln.201300196

[B134] Zhang X. Xing Y. Wang Q. Yan G. Wang M. Liu G.. (2020). Effects of long-term nitrogen addition and decreased precipitation on the fine root morphology and anatomy of the main tree species in a temperate forest. For. Ecol. Manage. 455, 117664. doi: 10.1016/j.foreco.2019.117664

[B135] Zhou G. Zhou X. Nie Y. Bai S. H. Zhou L. Shao J. (2018). Drought induced changes in root biomass largely result from altered root morphological traits: evidence from a synthesis of global field trials. Plant Cell Environ. 41, 2589–2599. doi: 10.1111/pce.13356 29879755

[B136] Zhu H. Zhao J. Gong L. (2021). The morphological and chemical properties of fine roots respond to nitrogen addition in a temperate schrenk's spruce (*Picea schrenkiana*) forest. Sci. Rep. 11, 1–12. doi: 10.1038/s41598-021-83151-x 33589690 PMC7884734

