# Supplementary material for: Response strategies of fine root morphology of Cupressus funebris to the different soil environment
Source: Front Plant Sci. 2022 Dec 21;13:1077090. doi: 10.3389/fpls.2022.1077090 (PMC9811150; doi:10.3389/fpls.2022.1077090)
Supplement: Supplementary file 1 [file DataSheet_1.doc]

**Supplementary tables**

| **Table S1 ANOVA of effects of site and root order on fine root morphology (*p-*value)** | | | | |
| --- | --- | --- | --- | --- |
| Source of variation | SRL | SRA | D | RTD |
| Site | <0.05 | <0.05 | <0.05 | <0.05 |
| Root order | <0.05 | <0.05 | <0.05 | <0.05 |
| Site × Root order | <0.05 | <0.05 | >0.05 | >0.05 |

Note: D: diameter; SRL: specific root length; SRA: specific surface area; RTD: root tissue density.

**Table S2** Soil physicochemical properties in four different research sites (mean ± SE)

| Soil index | GA | DY | SN | MY | *P-*value |
| --- | --- | --- | --- | --- | --- |
| Alkaline nitrogen (mg·kg^−1^) | 84.95±2.62b | 120.90±4.47d | 68.87±2.96a | 96.57±3.29c | 0.000** |
| Available phosphorus (mg·kg^−1^) | 6.27±0.09a | 7.47±0.31b | 6.27±0.31a | 6.40±0.26a | 0.031* |
| Organic carbon (g·kg^−1^) | 26.59±0.23d | 23.40±0.54c | 13.21±0.24a | 17.83±0.56b | 0.000** |
| Total nitrogen (g·kg^−1^) | 1.13±0.05a | 1.43±0.04b | 1.18±0.04a | 1.21±0.03a | 0.005** |
| Total phosphorus (g·kg^−1^) | 0.39±0.02ab | 0.41±0.00b | 0.36±0.00a | 0.39±0.01ab | 0.111^NS^ |
| Soil moisture (%) | 26.98±0.15d | 15.67±0.27a | 21.67±0.34c | 19.18±0.13b | 0.000** |
| Soil temperature (°C) | 17.68±0.09a | 20.21±0.06b | 21.30±0.03c | 21.79±0.05d | 0.000** |
| Soil bulk density (g.cm^−3^) | 1.15± 0.06a | 1.28±0.04ab | 1.56±0.06bc | 1.41±0.03c | 0.002** |
| Soil porosity (%) | 48.12±0.52a | 45.96±1.13b | 39.94±1.14bc | 43.06±0.70c | 0.001** |

Note: Different lowercase letters represent significant differences in the same soil index at different sites (*P <* 0.05). GA: Guangan; DY: Deyang; SN: Suining; MY: Mianyang. *Significant difference; **Extremely significant difference; NS, no significant difference.

**Table S3 List of abbeviations**

| Full Name | Abbreviations | Full Name | Abbreviations |
| --- | --- | --- | --- |
| Suining | SN | Soil bulk density | SBD |
| Deyang | DY | Soil porosity | SP |
| Mianyang | MY | Organic carbon | OC |
| Guangan | GA | Available phosphorus | AP |
| Diameter | D | Alkaline nitrogen | AN |
| Specific root length | SRL | Total nitrogen | TN |
| Specific surface area | SRA | Soil temperature | ST |
| Root tissue density | RTD | Total phosphorus | TP |
| Soil moisture | SW | Change rate | CR |
