# Supplementary material for: Response strategies of fine root morphology of Cupressus funebris to the different soil environment
Source: Front Plant Sci. 2022 Dec 21;13:1077090. doi: 10.3389/fpls.2022.1077090 (PMC9811150; doi:10.3389/fpls.2022.1077090)

Supplementary Figure

Figure S1 This picture of root order grading is taken from another article (Pregitzer et al., 2002).

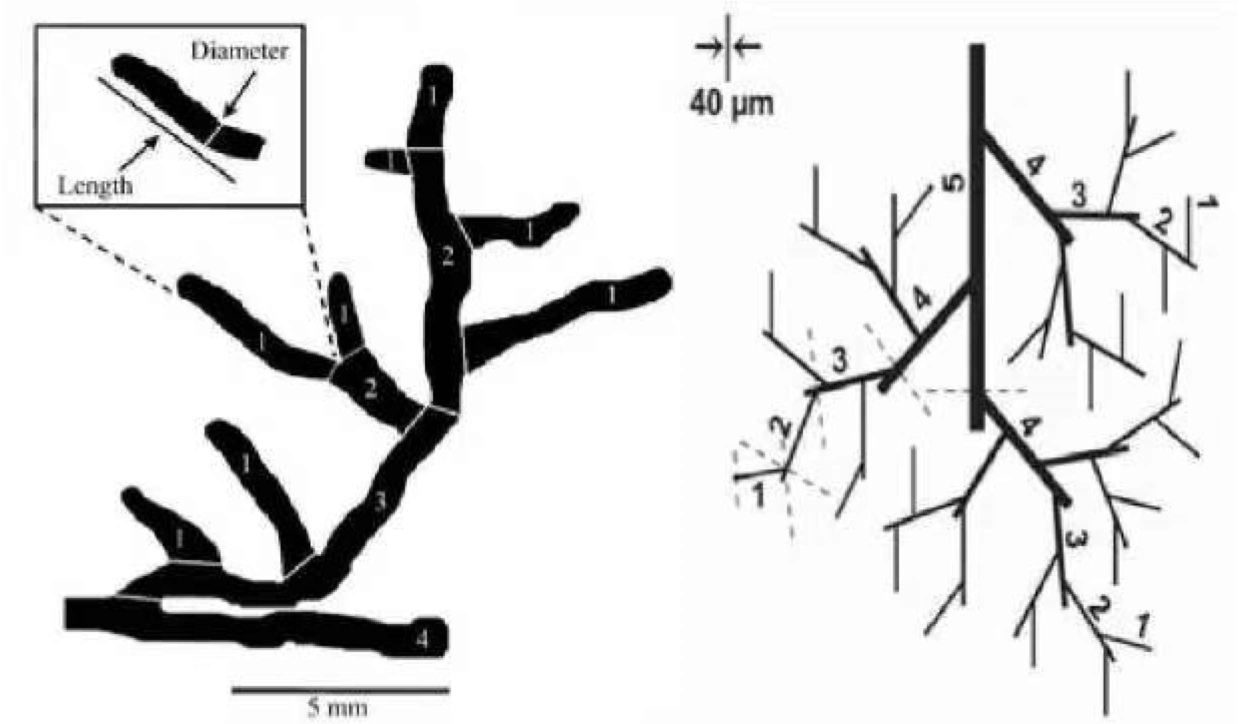

Supplement: Supplementary file 2 [file Presentation_1.pdf]
